# Antimicrobial, Quorum Sensing Inhibition, and Anti-Cancer Activities of Silver Nanoparticles Synthesized from Kenyan Bacterial Endophytes of *Teclea nobilis*

**DOI:** 10.3390/ijms26073306

**Published:** 2025-04-02

**Authors:** Farzana Mohamed, Hafizah Yousuf Chenia

**Affiliations:** Discipline of Microbiology (Westville Campus), School of Life Sciences, College of Agriculture, Engineering and Science, University of KwaZulu-Natal, Private Bag X54001, Durban 4000, KwaZulu-Natal, South Africa; 213505739@stu.ukzn.ac.za

**Keywords:** anti-cancer potential, antimicrobial activity, endophytic bacteria, quorum sensing inhibition, silver nanoparticles

## Abstract

Untapped bioactive compounds from microbial endophytes offer a promising solution to counter antimicrobial and chemotherapeutic drug resistance when complexed as silver nanoparticles (AgNPs). AgNPs were biosynthesized using cell-free supernatants from endophytic *Streptomyces* sp. KE4D and *Bacillus safensis* KE4K isolated from the Kenyan medicinal plant *Teclea nobilis*, following fermentation in three different media. Bacterial extracts were analyzed using gas chromatography–mass spectrometry. AgNPs were characterized using Fourier-transform infrared spectroscopy and high-resolution transmission electron microscopy. Antimicrobial activity was assessed using agar well diffusion assays, and quorum sensing inhibition (QSI) was investigated using *Chromobacterium violaceum*. Anti-cancer potential was evaluated against breast (MCF-7) and prostate cancer (DU-145) cell lines using MTT assays. AgNPs were 5–55 nm in size, with KE4D AgNPs being spherical and KE4K AgNPs exhibiting various shapes. Cyclopropane acetic acids and fatty acids were identified as possible capping agents. Medium-dependent antimicrobial activity was observed, with medium Mannitol and medium 5294 AgNPs displaying stronger activity, particularly against Gram-negative indicators. KE4D medium 5294 AgNPs demonstrated 85.12% violacein inhibition at 140 µg/mL and better QSI activity, whilst KE4K AgNPs were better antimicrobials. The AgNPs IC_50_ values were <3.5 µg/mL for MCF-7 and <2.5 µg/mL for DU-145 cells. The bioactivity of biosynthesized AgNPs is influenced by the bacterial isolate and fermentation medium, suggesting that AgNP synthesis can be tailored for specific bioactivity.

## 1. Introduction

The global rise of antimicrobial and chemotherapeutic drug resistance presents a critical challenge [1]. This growing threat underscores the urgent need for innovative, multi-disciplinary therapeutic solutions. Traditional medicine has long been used globally across various cultures, leading to the exploration of plants for bioactive phytochemicals with therapeutic potential [2]. While plants produce antimicrobial, antioxidant, and anti-inflammatory compounds, their association with endophytic microbes may significantly influence these bioactive properties [2].

Endophytes found in medicinal plants are rich sources of bioactive secondary metabolites that play important roles in signaling, defense, and plant–endophyte symbiosis [3,4]. The induction of a defense response by the endophyte is a direct result of secreted cumulative defense compounds. As a result, endophytes produce compounds with therapeutic potential, not only to support the host but to outcompete invading microbes in the plant [5]. Due to their remarkable species diversity and adaptability to various environments, endophytes serve as valuable reservoirs of bioactive compounds. These metabolites exhibit a wide range of biological activities, including antibacterial, anti-cancer, antifungal, anti-inflammatory, antioxidant, anti-quorum sensing, anti-viral, immunomodulatory, and hepatoprotective effects [1]. Despite their immense potential, endophytes remain an underexplored resource. Given the limited pipeline of new drugs to combat resistant infections and diseases, their metabolic diversity holds significant promise for pharmaceutical advancements.

To enhance the bioavailability and bioactivity of natural product therapies, researchers are exploring the functionalization of metallic nanoparticles (NPs) using endophytic biomolecules. These biomolecules prevent NP agglomeration and facilitate stabilization during synthesis. Due to their unique physiochemical properties, such as high reactivity, small size, and large surface areas with free dangling bonds, NPs offer significant advantages over their bulk counterparts [6]. However, conventional NP synthesis often involves toxic chemicals, highlighting the need for eco-friendly alternatives [7].

Biological synthesis of NPs offers a greener approach by reducing the release of toxic by-products [7]. Microorganisms serve as natural nano-factories, capable of detoxifying and reducing heavy metals through cellular reductase enzymes [8,9]. Amongst microbial NP synthesis methods, extracellular synthesis using cell-free supernatant (CFS) is particularly advantageous. It streamlines the process by eliminating the complex downstream recovery steps required for intracellular synthesis [10,11,12].

Endophyte-mediated silver nanoparticles (AgNPs) have demonstrated antimicrobial activity, effectively targeting pathogenic microorganisms [13]. Microbial survival in harsh environments and biofilm-associated resistance is regulated by quorum sensing (QS), a complex chemical signaling pathway [14]. Notably, bacterial biosynthesized AgNPs have demonstrated anti-QS potential, offering a promising strategy to combat bacterial biofilm resistance and pathogenicity [15,16]. Unlike conventional antibiotics, this approach attenuates virulence without killing the pathogens, reducing selective pressure and minimizing resistance development.

Interestingly, endophytic microbes produce an arsenal of QS-inhibitory small molecules and/or enzymes [17], making AgNPs an emerging tool for developing nanomaterials that target QS-regulated virulence factors. Beyond antimicrobial applications, AgNPs have also demonstrated significant potential in cancer therapy research [18,19,20,21,22]. Current anti-cancer treatments are often toxic, causing severe side effects and contributing to drug resistance [23]. Studies suggest that AgNPs may selectively target cancer cells by disrupting their ultrastructure, generating reactive oxygen species (ROS), inducing DNA damage, and ultimately triggering apoptosis and necrosis [24,25,26,27]. Given these promising findings, integrating endophyte-derived secondary metabolites with NP biosynthesis presents exciting opportunities for novel therapeutic applications [28].

While the antimicrobial potential of medicinal plants and their biosynthesized AgNPs has been extensively studied, limited research exists on the quorum sensing inhibition (QSI) and anti-cancer potential of AgNPs biosynthesized using endophytic bacteria. This study aimed to address this gap by synthesizing AgNPs from the cell-free supernatants (CFS) of two Kenyan bacterial endophytes isolated from the medicinal plant *Teclea nobilis*. The endophytes were fermented using three different culture media, and the resulting AgNPs were characterized and evaluated for their antimicrobial, QSI, and anti-cancer bioactivities.

## 2. Results

### 2.1. Molecular Identification of Bacterial Isolates

Genomic DNA was isolated, and the 16S rRNA gene of ~1500 bp was amplified from the two bacterial isolates, KE4D and KE4K. Sequencing of the amplified 16S rRNA genes identified bacterial isolate KE4D as a *Streptomyces* species isolate and KE4K as *Bacillus safensis.* The 16S rRNA gene sequences were deposited in the NCBI database with GenBank Accession Numbers PQ113811 and PQ113812, respectively.

### 2.2. Chemical Characterization of Endophytic Bacteria Crude Extracts

Fourier-transform infrared spectroscopy analysis of the crude bacterial extracts revealed absorbance bands corresponding to various functional groups, including alkanes, amines, alkenes, aromatic compounds, alkyl amines, and hydroxyl groups (Table 1; Supplementary Figure S1). These bands indicate the presence of terpenoids and flavonoids, which were likely produced by the bacterial isolates during fermentation. Absorption peaks at 1640–1550 cm^−1^ range are similar to those reported for proteins (Supplementary Figure S1). Additionally, peaks between 3650 and 3200 cm^−1^ suggest the presence of alcohol (O–H bands) groups, which may originate from proteins and carbohydrates in the crude extracts.

Gas chromatography–mass spectrometry proved to be a useful tool for identifying compounds in bacterial extract (Table 2, Table 3 and Table 4). Detailed compound information for bacterial extracts fermented in three different media (medium Mannitol, 5294, and 5333) can be found in Supplementary Tables S1–S6, together with the media controls (Supplementary Tables S7–S9). The chromatograms varied depending on the fermentation medium, with the highest compound diversity observed in *B. safensis* KE4K grown in medium Mannitol (Table 2) and *Streptomyces* sp. KE4D grown in medium 5294 (Table 3). Some of these compounds may act as metabolites with potential roles as capping agents during AgNP synthesis.

For *Streptomyces* sp. KE4D extracts, eicosane and eicosanoic fatty acid were consistently produced across all three media (Table 2, Table 3 and Table 4). However, differences in compound concentrations were observed. For example, while the bioactive compound pyrrolo[1,2-a]pyrazine-1,4-dione, hexahydro-3-(2)- was detected in all three media, its highest concentration (35.08%) was found in the medium Mannitol extract (Table 2). The greatest compound diversity was observed in the *Streptomyces* sp. KE4D extract from medium 5294 (Table 3).

For the *B. safensis* KE4K extracts, a greater diversity of compounds was observed in the medium Mannitol extract compared to the medium 5294 and 5333 extracts. The dominant compounds in the medium Mannitol extract included isovaline, 3-hydroxy- (24.773%), 2-(2-hydroxy-2-methyl-3-oxobutyl)-3,5,6-trimethylpyrazine (2.61%), and 3-amino-3-(4-isopropoxy-phenyl)-propionic acid (8.30%). Pyrazine and tetramethyl- were the only compounds present in both medium Mannitol (6.61%) and medium 5294 (4.10%) extracts at high concentrations. In contrast, the dominant compounds in the medium 5333 extracts (Table 4) were tricosane-2,4-dione (18.48%) and diisooctyl phthalate (17.17%).

### 2.3. Silver Nanoparticle Biosynthesis

**Figure 1:**
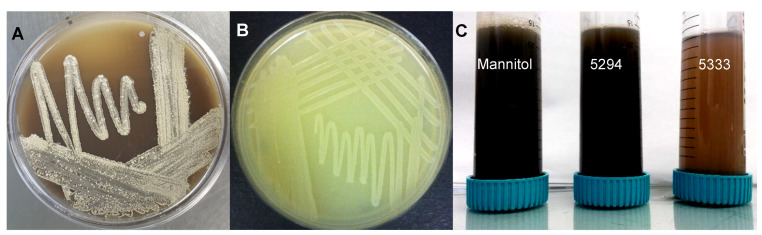
Endophytic bacteria (**A**) *Streptomyces* sp. KE4D and (**B**) *Bacillus safensis* KE4K, isolated from *Teclea nobilis,* were selected for silver nanoparticle (AgNP) synthesis. (**C**) Color change of the bacterial cell-free supernatant and silver nitrate reaction mix from clear to grey-black was indicative of AgNP formation. For *B. safensis* KE4K AgNPs synthesized from medium Mannitol and medium 5294, a dark black reaction mix was observed, whilst for *B. safensis* KE4K medium 5333 AgNPs, the reaction mix appeared light brown.

Silver nitrate (AgNO_3_) was reduced to elemental silver (Ag^+^) upon its addition to the bacterial CFS (Figure 1C), as indicated by the formation of a grey-black reaction mixture after incubation. Among the fermentation media, cultures grown in medium Mannitol produced the highest biomass, followed by those in medium 5294 and 5333. Similarly, AgNP synthesis was most efficient in the CFS from medium Mannitol fermentation followed by medium 5294, whereas little to no AgNPS were observed with the CFS of medium 5333. When a sterile medium was incubated with AgNO_3_, no grey-black color change or pellet formation occurred, suggesting that AgNO_3_ did not reduce to Ag^+^ in the absence of bacterial metabolites.

### 2.4. Characterization of Nanoparticles

#### 2.4.1. UV-Visible Spectroscopy Confirmation of Nanoparticle Synthesis

**Figure 2:**
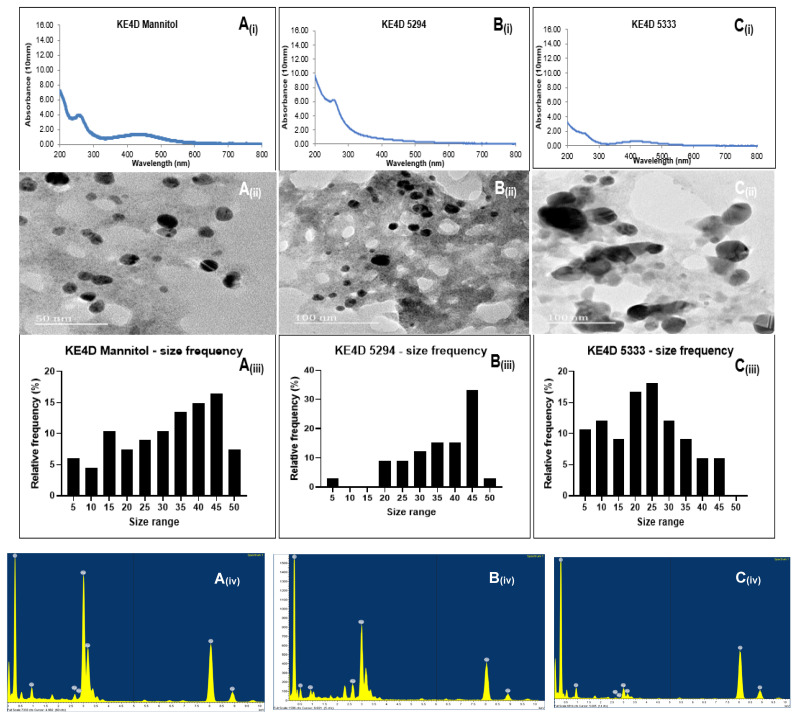
UV-visible spectroscopy results of silver nanoparticles (AgNPs) biosynthesized from (**A_(i)_**) endophytic *Streptomyces* sp. KE4D medium Mannitol, (**B_(i)_**) *Streptomyces* sp. KE4D medium 5294, and (**C_(i)_**) *Streptomyces* sp. KE4D medium 5333 indicating a peak between 200 and 300 nm, which is characteristic of AgNPs. High-resolution transmission electron micrographs showed that AgNPs from (**A_(ii)_**) *Streptomyces* sp. KE4D medium Mannitol, (**B_(ii)_**) *Streptomyces* sp. KE4D medium 5294, and (**C_(ii)_**) *Streptomyces* sp. KE4D medium 5333 were spherical. Size distribution analysis of (**A_(iii)_**) *Streptomyces* sp. KE4D medium Mannitol, (**B_(iii)_**) *Streptomyces* sp. medium KE4D 5294, and (**C_(iii)_**) *Streptomyces* sp. KE4D medium 5333 indicated AgNPs were in the 5–50 nm range. Using energy-dispersive X-ray analysis (**A_(iv)_**–**C_(iv)_**), Ag in the 3 keV region was observed as the predominant elemental constituent for *Streptomyces* sp. KE4D AgNPs.

**Figure 3:**
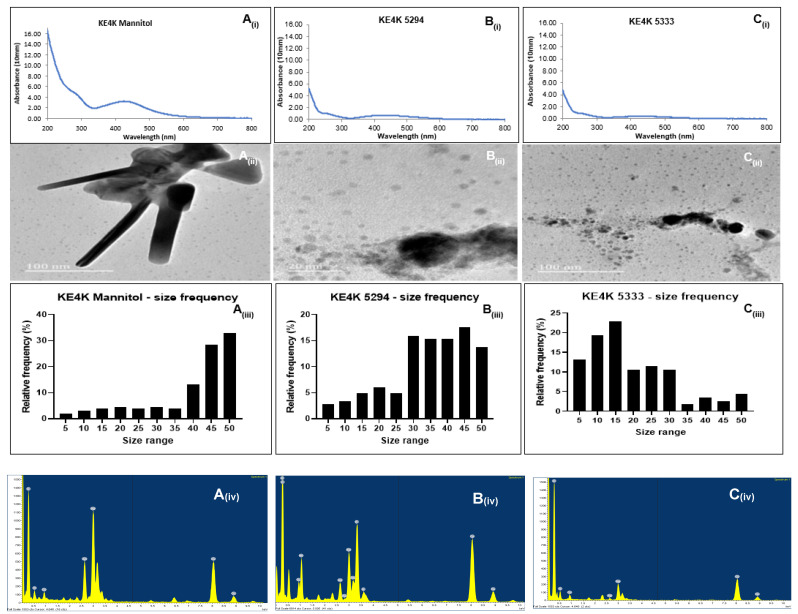
UV-visible spectroscopy results of silver nanoparticles (AgNPs) biosynthesized from (**A_(i)_**) endophytic *Bacillus safensis* KE4K medium Mannitol, (**B_(i)_**) *B. safensis* KE4K medium 5294, and (**C_(i)_**) *B. safensis* KE4K medium 5333 indicating a peak between 200 and 500 nm, which is characteristic for AgNPs. High-resolution transmission electron micrographs showed that AgNPs from (**A_(ii)_**) *B. safensis* KE4K medium Mannitol were spherical-, rod-, and triangular-shaped, and (**B_(ii)_**) *B. safensis* KE4K medium 5294 and (**C_(ii)_**) *B. safensis* KE4K medium 5333 were spherical. Size distribution analysis of (**A_(iii)_**) *B. safensis* KE4K medium Mannitol, (**B_(iii)_**) *B. safensis* KE4K medium 5294, and (**C_(iii)_**) *B. safensis* KE4K medium 5333 indicated AgNPs were in the 5–55 nm range. Using energy-dispersive X-ray analysis (**A_(iv_**_)_–**C_(iv)_**), Ag in the 3 keV region was observed as the predominant elemental constituent for *B. safensis* KE4K AgNPs.

The reduction of AgNO_3_ to Ag^+^ was confirmed using the Nanophotometer NP80 (Implen), which detected plasmon resonance peaks in the visible light region of 200–500 nm (Figure 2A_(i)_–C_(i)_ and Figure 3A_(i)_–C_(i)_). Since silver is known to have plasmon resonance peaks in the 400–800 nm range [29], the metal was confirmed to be silver. Higher absorbances were observed for medium Mannitol AgNPs, followed by medium 5333 and 5294 AgNPs. This suggests a higher concentration of capping agents on AgNPs from medium Mannitol and medium 5333 compared to those from medium 5294. Broad plasmon peaks for AgNPs synthesized from medium Mannitol and medium 5294 indicate an increase in particle size. In contrast, narrow peaks observed between 200 and 300 nm for KE4D Mannitol and 5333 AgNPs are likely due to interference from microbial constituents, such as polyphenols in the media [30,31].

#### 2.4.2. Fourier-Transform Infrared Spectroscopy Analysis

Fourier-transform infrared spectroscopy was employed to identify the biochemical interactions between the biosynthesized AgNPs and the biomolecules present in the CFS. For *Streptomyces* sp. KE4D AgNPs, which were synthesized using CFS from three different fermentation media, similar absorption spectra were observed in the 2400 cm^−1^ to 800 cm^−1^ range (Table 1; Supplementary Figure S2). Common functional groups included alkynes (C≡C), strong isothiocyanates (N=C=S), and strong nitro groups (N-O) (Table 1), suggesting that bacterial organic molecules played a role in the silver ion reduction and NP stabilization [32]. Isothiocyanates, which are bioactive compounds derived from glucosinolate hydrolysis, are also known for their antibacterial and anti-cancer properties [33]. In the 3800 cm^−1^ to 2400 cm^−1^ range, distinct spectral patterns were observed for KE4D AgNPs, indicating variations in the biochemical environment. The persistence of negatively charged hydroxyl (O-H) groups in the *Streptomyces* sp. KE4D medium Mannitol and medium 5294 AgNPs suggest enhanced silver ion reduction and the presence of flavonoid and phenol groups [34]. Additionally, functional groups associated with phenazine compounds, which are broad-spectrum antimicrobial metabolites produced by *Streptomyces* species [34], typically form peaks between 3400 cm^−1^ and 3000 cm^−1^. These peaks were observed in *Streptomyces* sp. KE4D AgNPs synthesized from medium 5294 and medium 5333.

Silver nanoparticles synthesized from *B. safensis* KE4K CFS exhibited similar spectral patterns for medium Mannitol and medium 5294 AgNPs (Table 1; Supplementary Figure S2). Common functional groups included strong hydroxyl (O-H) (3600–3400 cm^−1^), strong amine salt (N-H) (3000–2800 cm^−1^), and weak bending of aromatic (C-H) compounds (2000–1800 cm^−1^). The peaks in the 2000 cm^−1^ to 1800 cm^−1^ range indicate the presence of aromatic structures typical of flavonoids, which are characterized by a C6–C3–C6 phenyl benzopyran structure [34,35]. Peaks between 500 cm^−1^ and 1500 cm^−1^ represent carbohydrate fingerprints. In contrast, a distinct spectral pattern was observed for *B. safensis* KE4KAgNPs synthesized from medium 5333. These exhibited a medium sharp hydroxyl (O-H) stretch in the 3800 cm^−1^ to 3600 cm^−1^ region, likely due to the presence of phenolic or flavonoid compounds [36]. Weak nitrile (C≡N) bonds were detected in the 2400 cm^−1^ to 2200 cm^−1^ region, while weak bending of aromatic (C-H) compounds appeared in the 1200 cm^−1^ to 1000 cm^−1^ region. These findings suggest that AgNPs contain bacterial-derived compounds with nitrile and aromatic structures [37].

Among all AgNPs, the most common functional groups identified were hydroxyl, alkyne, isothiocyanate, and nitro compounds (Table 1). The presence of alkyne groups can be attributed to organic molecules secreted by the bacteria, which play a role in the reduction and stabilization of AgNP [38]. Hydroxyl groups are typically associated with phenolic and flavonoid compounds, which enhance the reduction of Ag^+^ to Ag^0^ [22]. Additionally, isocyanates, derived from glucosinolates produced by plants and microorganisms, contribute to the stabilization and potential biological activity of AgNPs by forming strong interactions with the NP surface [39,40]. The FTIR analysis of KE4D and KE4K AgNPs suggests that biological compounds from the bacterial CFS formed strong capping agents on the AgNPs, contributing to their stability.

#### 2.4.3. High-Resolution Transmission Electron Microscopy Characterization

The morphology and sizes of the biosynthesized AgNPs were determined using HR-TEM (Figure 2A_(ii)_–C_(ii)_ and Figure 3A_(ii)_–C_(ii)_; Table 5). Across all three fermentation media, AgNPs synthesized from both *Streptomyces* sp. KE4D and *B. safensis* KE4K were predominantly spherical. However, AgNPs synthesized from *B. safensis* KE4K medium Mannitol displayed triangular and rod-shaped AgNPs (Figure 3A_(ii)_).

For *Streptomyces* sp. KE4D, AgNPs from medium Mannitol (Figure 2A_(ii)_) appeared clustered and spherical, while those from medium 5294 (Figure 2B_(ii)_) were spherical and non-aggregated. Smaller, non-aggregated AgNPs were observed for *Streptomyces* sp. KE4D AgNPs from medium 5333 (Figure 2C_(ii)_). In contrast, *B. safensis* KE4K AgNPs synthesized from medium Mannitol (Figure 3A_(ii)_) were aggregated and displayed triangular and rod-shaped structures. Smaller, less aggregated *B. safensis* KE4K AgNPs were observed with medium 5294 (Figure 3B_(ii)_) and medium 5333 (Figure 3C_(ii)_).

Image analysis of the AgNPs indicated that their size was influenced by the fermentation medium. AgNPs synthesized from medium Mannitol had larger sizes, followed by those synthesized from medium 5294 and medium 5333 (Figure 2A_(iii)_–C_(iii)_ and Figure 3A_(iii)_–C_(iii)_; Table 5). The overall size range of the AgNPs was between 5 and 55 nm (Figure 2A_(iii)_–C_(iii)_ and Figure 3A_(iii)_–C_(iii)_). Size distribution analysis of *Streptomyces* sp. KE4D AgNPs indicated that 21.76% of medium Mannitol AgNPs (Figure 2A_(iii)_) measured between 45 and 49 nm were in the same size range, while 27.65% of medium 5294 AgNPs (Figure 2B_(iii)_) were in the same size range. In contrast, 26.47% of medium 5333 AgNPs (Figure 2C_(iii)_) were smaller when measuring between 15 and 19 nm. Similarly, for *B. safensis* KE4K AgNPs, 22.35% of KE4K medium Mannitol AgNPs (Figure 3A_(iii)_) were between 50 and 55 nm, while 23.53% of medium 5294 AgNPs (Figure 3B_(iii)_) were 45–49 nm, and 18.82% of medium 5333 AgNPs (Figure 3C_(iii)_) were between 15 and 19 nm.

#### 2.4.4. Zeta Potential Determination

The average charge of the biosynthesized AgNPs was determined using the DLS technique. Most of the biosynthesized AgNPs were predominantly negatively charged, except for *B. safensis* KE4K medium Mannitol AgNPs, which were positively charged (Table 5). All *Streptomyces* sp. KE4D and *B. safensis* KE4K AgNPs, regardless of the fermentation medium, exhibited zeta potentials within the delicate dispersion threshold (−20 to −10 mV and 10 to 20 mV), indicating relative stability [41,42].

### 2.5. Antimicrobial Activity Testing

The antimicrobial activity of the *Streptomyces* sp. KE4D (Supplementary Figure S3) and *B. safensis* KE4K (Supplementary Figure S4) AgNPs was tested against *A. baumannii* ATCC 19606, β-lactam resistant *E. coli* ATCC 35218, vancomycin-resistant *E. faecalis* ATCC 29212, *L. monocytogenes* ATCC 19111, multidrug-resistant *P. aeruginosa* ATCC 27853, and MRSA ATCC 43300.

The AgNPs exhibited stronger antimicrobial activity against Gram-negative bacteria compared to Gram-positive bacteria (Table 6). *B. safensis* KE4K AgNPs from medium Mannitol and medium 5294 displayed strong antimicrobial activity (≥16 mm inhibition zone) against *E. faecalis*. *Streptomyces* sp. KE4D AgNPs from medium Mannitol exhibited intermediate activity, while weak activity was observed with *Streptomyces* sp. KE4D AgNPs from medium 5294 and medium 5333.

For *A. baumannii*, *L. monocytogenes*, and *P. aeruginosa*, most *Streptomyces* sp. KE4D and *B. safensis* KE4K AgNPs demonstrated weak to intermediate antimicrobial activity. However, promising antimicrobial activity was observed against MRSA ATCC 43300, with strong inhibition by *Streptomyces* sp. KE4D medium 5294 and *B. safensis* KE4K medium Mannitol AgNPs. *Streptomyces* sp. KE4D medium 5294 AgNPs also exhibited strong antimicrobial activity against *E. coli*.

Overall, *Streptomyces* sp. KE4D AgNPs demonstrated broader antimicrobial activity compared to *B. safensis* KE4K AgNPs (Table 6). In particular, *Streptomyces* sp. KE4D medium 5294 and KE4K medium Mannitol AgNPs demonstrated promising antimicrobial potential against both Gram-positive and Gram-negative bacteria.

### 2.6. Quorum Sensing Inhibitory Potential

The QSI activity of AgNPs (100–200 µg/mL) synthesized from *Streptomyces* sp. KE4D and *B. safensis* KE4K was evaluated using *C. violaceum* ATCC 12472 as the indicator organism (Supplementary Figure S5). Good QSI was defined as AgNPs exhibiting ≥50% VI and <40% GI (Table 7). Both *Streptomyces* sp. KE4D and *B. safensis* KE4K AgNPs demonstrated better QSI compared to the control vanillin, which required concentrations above 400 µg/mL to achieve >50% QSI (Table 7).

Among the three sets of *Streptomyces* sp. KE4D AgNPs, only those synthesized in medium Mannitol and medium 5294 exhibited ≥ 50% QSI (Table 7). *Streptomyces* sp. KE4D medium 5294 AgNPs were the most effective, achieving 85.12% VI at 140 µg/mL, though they were bactericidal at ≥160 µg/mL (Table 7; Supplementary Figure S5B).

In comparison, *B. safensis* KE4K AgNPs synthesized from medium Mannitol and medium 5294 demonstrated good QSI activity, with 55.94% and 50%, respectively, at 140 µg/mL but also exhibited bactericidal effects at 160 µg/mL (Table 7; Supplementary Figure S5D,E). While *B. safensis* KE4K medium 5333 AgNPs achieved good VI at 160 µg/mL, they became bactericidal at ≥180 µg/mL (Supplementary Figure S5F). The effects of AgNP concentration and fermentation medium on %VI and %GI at 140–200 μg/mL were found to be statistically significant *(p* ≤ 0.01) for both *Streptomyces* sp. KE4D and *B. safensis* KE4K AgNPs.

### 2.7. Cytotoxicity Assessment Using MTT Assay

**Figure 4:**
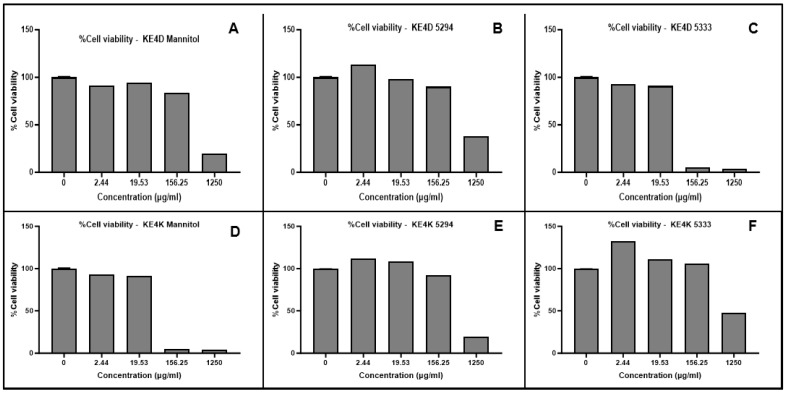
MTT cytotoxicity results depicting the dose-dependent cytotoxic effects of endophytic *Streptomyces* sp. KE4D (**A**–**C**) and *Bacillus safensis* KE4K (**D**–**F**) silver nanoparticles (AgNPs), biosynthesized from cell-free supernatants obtained following fermentation in three different media (medium Mannitol, 5294 and 5333), against the breast cancer MCF-7 cell line. Data represent the mean of two independent experiments performed in triplicate.

**Figure 5:**
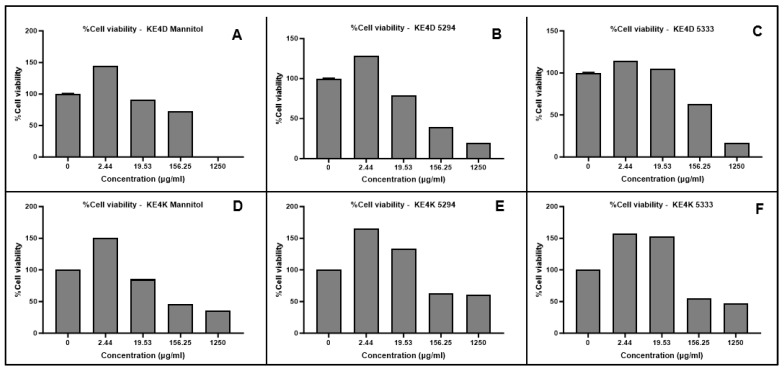
MTT cytotoxicity results depicting the dose-dependent cytotoxic effects of endophytic *Streptomyces* sp. KE4D (**A**–**C**) and *Bacillus safensis* KE4K (**D**–**F**) silver nanoparticles (AgNPs), biosynthesized from cell-free supernatants obtained following fermentation in three different media (medium Mannitol, 5294 and 5333), against the prostate cancer DU-145 cell line. Data represent the mean of two independent experiments performed in triplicate.

**Figure 6:**
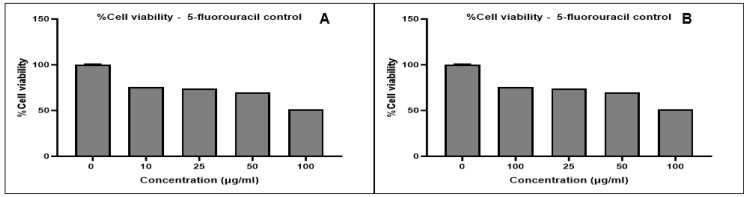
MTT cytotoxicity results for the 5-fluorouracil control against the (**A**) breast cancer MCF-7 and (**B**) prostate cancer DU-145 cell lines. Data represent the mean of two independent experiments performed in triplicate.

**Figure 7:**
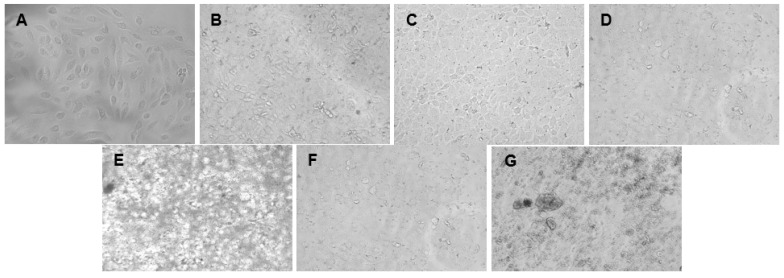
Bright-field microscopy images (20× magnification) of the cell morphology of the breast cancer MCF-7 cell line following treatment with endophytic *Streptomyces* sp. KE4D and *Bacillus safensis* KE4K silver nanoparticles (AgNPs) at 156.25 µg/mL: (**A**) untreated MCF-7 cells, (**B**) *Streptomyces* sp. KE4D medium Mannitol, (**C**) *Streptomyces* sp. KE4D medium 5294, (**D**) *Streptomyces* sp. KE4D medium 5333 AgNPs, (**E**) *B. safensis* KE4K medium Mannitol, (**F**) *B. safensis* KE4K medium 5294, and (**G**) *B. safensis* KE4K medium 5333 AgNPs. A high degree of cellular disintegration was observed between 156.25 and 1250 µg/mL.

**Figure 8:**
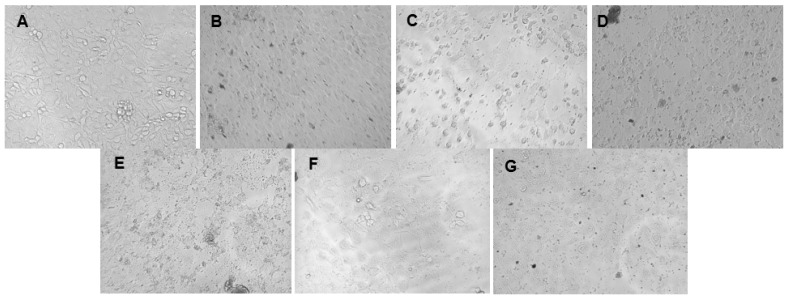
Bright-field microscopy images (20× magnification) of the cell morphology of the prostate cancer DU-145 cell line following treatment with endophytic *Streptomyces* sp. KE4D and *Bacillus safensis* KE4K silver nanoparticles (AgNPs) at 156.25 µg/mL: (**A**) untreated DU-145 cells, (**B**) *Streptomyces* sp. KE4D medium Mannitol, (**C**) *Streptomyces* sp. KE4D medium 5294, (**D**) *Streptomyces* sp. KE4D medium 5333, (**E**) *B. safensis* KE4K medium Mannitol, (**F**) *B. safensis* KE4K medium 5294, and (**G**) *B. safensis* KE4K medium 5333. A high degree of cellular disintegration was observed between 156.25 and 1250 µg/mL.

The cytotoxicity of the green synthesized AgNPs was evaluated against breast cancer MCF-7 and prostate cancer DU-145 cell lines. A dose-dependent decrease in cell viability was observed for both cell lines with increasing AgNP concentrations (Figure 4 and Figure 5) and for the positive control, 5-fluorouracil (Figure 6). Morphological changes visualized using bright-field microscopy (20× magnification; EVOS^®^ FL Cell Imaging System; Thermo Fischer Scientific, Waltham, MA, USA) revealed progressive cell disintegration at higher AgNP concentrations (Figure 7 and Figure 8).

Prostate cancer DU-145 cells exhibited greater sensitivity to AgNPs compared to breast cancer MCF-7 cells, as indicated by higher cytotoxicity and reduced viability and proliferation. All AgNPs demonstrated significant cytotoxic potential (*p ≤* 0.0001) at varying concentrations against both cell lines when compared to 5-fluorouracil. While differences in IC_50_ values were observed based on the fermentation medium used for AgNP synthesis, these differences were not significant.

The IC_50_ values for AgNPs were determined after normalization of the absorbance values and transformation of the AgNP concentrations using GraphPad Prism 10 (Table 8). Overall, AgNPs exhibited greater efficacy against prostate cancer DU-145 cells compared to breast cancer MCF-7 cells. While not statistically significant, *Streptomyces* sp. KE4D AgNPs displayed higher cytotoxic activity against both cell lines than *B. safensis* KE4K AgNPs. The lowest IC_50_ value against MCF-7 was observed for *Streptomyces* sp. KE4D medium 5333 AgNPs (IC_50_ = 2.034 µg/mL), whereas *B. safensis* KE4K medium Mannitol AgNPs exhibited the lowest IC50 against the DU-145 cell line (IC_50_ = 1.938 µg/mL). Compared to the positive control, 5-fluorouracil (MCF-7 IC_50_ = 21.75 µg/mL; DU-145 IC_50_ = 43.02 µg/mL), AgNPs synthesized from *Teclea nobilis* bacterial endophytes demonstrated significantly lower IC_50_ values. This suggests that these AgNPs could serve as promising anti-cancer agents, given their potent cytotoxic effects at low dosages.

## 3. Discussion

Bioprospecting natural environments have garnered significant interest in the pursuit of novel compounds to address current medical challenges. The Kakamega rainforest, from which the endophytic bacteria have been isolated, has been identified as a valuable reservoir of medicinal plants with therapeutic potential [43]. However, the unsustainable harvesting of medicinal plants, which disrupts ecosystems, remains a fundamental issue [44]. For this reason, exploring endophytic bacteria for medicinal application offers distinct advantages, as it eliminates the need for habitat destruction, helping to preserve biodiversity and prevent plant extinction [45]. The use of endophytes in pharmaceutical research is rapidly gaining momentum due to their capacity to synthesize novel secondary metabolites with antimicrobial, antioxidant, and anti-cancer properties [46]. However, translating these natural compounds into clinical applications has been hindered by their poor bioavailability [47]. The integration of natural products with AgNPs enhances their targeted delivery, a benefit supported by the GC-MS analyses of the crude bacterial extracts in the present study. The combination not only improves targeting efficiency but also enhances bioactivity and stability [48,49,50].

In this study, AgNP synthesis was carried out using the CFS of two endophytic bacteria grown in three different fermentation media. The AgNP yield was directly proportional to biomass yield obtained following fermentation, with the highest production observed in medium Mannitol, followed by medium 5294 and medium 5333. Medium Mannitol, a nutrient-rich medium, contains D-Mannitol as the primary carbon source along with starch. Medium 5294 utilizes starch and glucose as the main carbon substrates, while medium 5333 is starch-based but also contains salts such as dipotassium phosphate and magnesium-reported sulfate monohydrate. Shaaban et al. [22] found that starch-containing media were the most preferred carbon source for the endophytic bacterium *Streptomyces enissocaesilis* BS1 in AgNP biosynthesis, which may explain the higher AgNP yield from medium Mannitol and medium 5294 in this study. Additionally, Martin [51] reported that phosphate inhibits the production of secondary metabolites, potentially contributing to lower yield observed for medium 5333 AgNPs. These findings further support the use of starch-based growth media for efficient AgNP biosynthesis.

Fourier-transform infrared spectroscopy is a valuable tool for characterizing the surface chemistry of AgNPs [52]. Bioactive compounds secreted by endophytes act as capping agents, enhancing the stability of the NPs [53]. Functional groups such as carbonyls, hydroxyls, and amines bind to the AgNP surface, facilitating stabilization [54]. In this study, these functional groups were identified in the FTIR spectra and GC-MS chromatograms of crude ethyl acetate extracts from endophytic bacteria (Table 1, Table 2, Table 3 and Table 4). FTIR analysis revealed dominant peaks corresponding to alkyne-, isothiocyanate-, and nitro-containing compounds across both *Streptomyces* sp. KE4D and *B. safensis* KE4K AgNPs (Table 1). Hydroxyl groups play a crucial role in AgNP stabilization and reduction, contributing to hydrogen bonding in secondary protein structures [55]. These groups are also key components of phenolic compounds, further enhancing NP [36]. The presence of isothiocyanate-containing compounds is important, as they not only stabilize AgNPs but also exhibit significant biological activity. Hać et al. [56] demonstrated that these compounds possess anti-cancer properties by inducing apoptosis, inhibiting cell proliferation, and disrupting cancer cell signaling pathways. Additionally, Richa et al. [57] reported their antibacterial activity, showing rapid efficacy against *E. coli* and *S. aureus* within 90 and 120 min, respectively. Similarly, nitro-containing compounds have been implicated in the capping and stabilization of green synthesized NPs, further supporting their multifunctional role in AgNP synthesis and bioactivity [58,59,60]. Collectively, these findings highlight the critical contribution of these functional groups to both the structural stability and biological efficacy of AgNPs.

GC-MS analysis of the *Streptomyces* sp. KE4D medium Mannitol extract identified cyclopropane acetic acid and 2-hexyl compounds (Table 2), consistent with previous reports of these metabolites in bacterial extracts [61,62]. The prominence of fatty acid groups in the *Streptomyces* sp. KE4D extract corresponds with its broad-spectrum antimicrobial activity against both Gram-positive and Gram-negative microorganisms. Although the exact mechanisms underlying fatty acid-mediated antimicrobial activity remain unclear, they are hypothesized to inhibit fatty acid desaturases [62]. Additionally, the KE4K medium Mannitol AgNPs exhibited a higher prevalence of amine groups compared to other AgNP samples (Table 1). Frattini et al. [63] demonstrated that amine groups play a crucial role in reducing Ag^+^ ions and forming stabilizing capping agents, which may explain the larger NP size observed for *B. safensis* KE4K medium Mannitol AgNPs.

The significant presence of hydroxyl groups identified in the FTIR analysis (Table 1) can be attributed to isobutyl isothiocyanate in the *B. safensis* KE4K medium 5294 crude extract. This compound exhibits diverse bioactive properties, including potent antimicrobial [64], antifungal [65], and anti-cancer [66] effects. The capping of the AgNPs with these endophytic bacterial extracts may act synergistically, where both the AgNPs and the isothiocyanate compounds contribute to the enhanced therapeutic efficacy [67]. Beyond stabilizing the nanoparticles, isobutyl isothiocyanate may improve their bioactivity and biocompatibility across various biomedical applications. Song et al. [68] reported that a rhodamine isothiocyanate analog from the coral bacterium *Vibrio alginolyticus* H12 exhibited QSI activity by down-regulating key regulatory genes in *Pseudomonas aeruginosa* PAO1. This highlights the potential of isothiocyanate compounds derived from *Bacillus* and *Streptomyces* species as an untapped resource for modulating antimicrobial activity, which could significantly influence therapeutic agent development.

Additionally, the bioactive diketopiperazine Cyclo(-Leu-Pro) or Pyrrolo[1,2-a]pyrazine-1,4-dione, hexahydro-3-(2-methylpropyl) was detected in high amounts in *Streptomyces* sp. KE4D medium Mannitol, medium 5294, and 5333 crude extracts but was found in negligible amounts in *B. safensis* KE4K medium 5294 and was absent in the medium Mannitol and medium 5333 extracts (Table 2, Table 3 and Table 4). Rajivgandhi et al. [69] demonstrated that this compound was isolated from *Nocardiopsis* sp. GRG 1 exhibited weak antibacterial activity but potent QSI and anti-biofilm effects against *Proteus mirabilis* and *E. coli*. Thus, its interaction with AgNPs may enhance both antibacterial and QSI efficacy, as evidenced by the QS inhibitory effect observed with *Streptomyces* sp. KE4D medium Mannitol and medium 5294 AgNPs (Table 7).

FTIR analyses further support that the reduction, stabilization, and dispersion of AgNPs are primarily driven by interactions with organic compounds from bacterial CFS. These compounds act as capping agents through various biochemical interactions, including hydroxyl isothiocyanate and amine group functionalities and negatively charged carboxyl groups from exogenous enzymes in CFS [70].

High-resolution transmission electron microscopy revealed that smaller-sized AgNPs were poly-dispersed, uniformly distributed, and exhibited minimal aggregation. In contrast, the larger *Streptomyces* sp. KE4D medium Mannitol (Figure 2A_(ii)_; Table 5) and *B. safensis* KE4K medium Mannitol (Figure 3A_(ii)_; Table 5) AgNPs, though aggregated, were not in direct contact with one another. This suggests the presence of a thin film, likely composed of capping agents, stabilizing the NPs and preventing fusion [71]. The shape of AgNPs plays a crucial role in their antimicrobial efficacy. Triangular-shaped NPs have been previously reported to exhibit stronger antimicrobial activity against both Gram-positive and Gram-negative microorganisms compared to their spherical counterparts [72,73]. This trend was also observed in the present study, where *B. safensis* KE4K medium Mannitol AgNPs displayed higher antimicrobial activity than those synthesized from medium 5333 and medium 5294 (Table 6). The enhanced antimicrobial potency of anisotropic triangular AgNPs is attributed to their {111} crystal facets, which exhibit higher surface reactivity and a greater number of atomic indices, thereby increasing their interaction with microbial cells [51,74,75]. Additionally, Ratan et al. [76] demonstrated that triangular silver nano-prisms with sharp edges and vertices exhibit superior antibacterial activity compared to spherical and near-spherical particles AgNPs. This enhanced activity is likely due to their ability to physically pierce bacterial cell membranes, causing structural damage and increased susceptibility to nanoparticle-induced cytotoxicity. These findings further underscore the importance of nanoparticle morphology in determining their biological efficacy.

Zeta potential analysis revealed that the biosynthesized AgNPs exhibited relative stability, aligning with the criteria established by Honary and Zahir [41]. Most AgNPs carried a negative charge, with values ranging from −12 to −17 mV, except for the *B. safensis* KE4K medium Mannitol AgNPs, which were positively charged. The presence of a positive charge on these AgNPs is particularly noteworthy, as positive NPs have been shown to enhance electrostatic attractions with negatively charged bacterial and tumor cell membranes, thereby increasing their bioactivity [77]. While the predominantly negative zeta potential (−12 to −17 mV) suggests a potential for aggregation, Joseph and Singhvi (77) emphasize that zeta potential alone is not a definitive measure of NP stability. Other key factors, such as NP composition, surface functionalization, and solution chemistry, play crucial roles in determining the overall stability of nanosuspensions [78,79]. Therefore, although zeta potential provides valuable insights into NP interactions, it should be evaluated in conjunction with additional stability parameters for a more comprehensive assessment.

The distinctive morphological characteristics and positive charge of *B. safensis* KE4K medium Mannitol AgNPs likely contributed to their heightened antimicrobial activity (Supplementary Figure S4; Table 6). The electrostatic interaction between positively charged AgNPs and the negatively charged bacterial cell membrane potentially facilitated NP adhesion, leading to membrane destabilization and permeability changes [80]. The biosynthesized *Streptomyces* sp. KE4D and *B. safensis* KE4K AgNPs exhibited greater antimicrobial activity against Gram-negative bacteria than Gram-positive bacteria. This aligns with previous findings suggesting that AgNPs tend to be more effective against Gram-negative bacteria than Gram-positive bacteria due to differences in cell wall composition despite varying resistance levels [81]. Overall, *Streptomyces* sp. KE4D AgNPs exhibited superior broad-spectrum antimicrobial activity compared to *B. safensis* KE4K AgNPs. This enhanced activity may be attributed to the presence of the antimicrobial compound Pyrrolo[1,2-a]pyrazine-1,4-dione, hexahydro-3-(2-methylpropyl) in *Streptomyces* sp. KE4D crude extracts. Rajivgandhi et al. [82] reported that Pyrrolo-containing compounds exert antimicrobial effects by inducing cellular leakage, disrupting bacterial morphology, and compromising bacterial membrane integrity. When combined with AgNPs, which adhere to and penetrate bacterial membranes while interacting with phosphorus-containing compounds such as DNA [83,84], a synergistic enhancement of antimicrobial efficacy is observed. Additionally, AgNPs are known to induce oxidative stress by generating ROS, such as superoxide radicals and hydroxyl radicals, which can damage bacterial proteins, lipids, and DNA, ultimately leading to cell death [82]. This effect was particularly evident with *Streptomyces* sp. KE4D AgNPs, as reflected in their superior antimicrobial performance (Table 6).

The 2018 listeriosis outbreak in South Africa sparked interest in evaluating the susceptibility of *Listeria monocytogenes* to the biosynthesized AgNPs. *Bacillus safensis* KE4K medium Mannitol AgNPs (Supplementary Figure S4E; Table 6) exhibited significant growth inhibition against *L. monocytogenes*, followed by AgNPs produced from *Streptomyces* sp. KE4D medium Mannitol and *Streptomyces* sp. KE4D medium 5333. Although the antimicrobial activity of the AgNPs was ranked as intermediate against *L. monocytogenes* (Table 6)*,* combining them with antibiotics may offer new avenues for the clinical treatment of *Listeria* infections.

Bioactive agents that interfere with QS have emerged as a promising class of antimicrobials that target bacterial virulence rather than bacterial viability [85]. Quorum sensing in *Chromobacterium violaceum* ATCC 12472 is primarily regulated by long-chain AHLs, which bind to CviR receptors and activate the transcription of violacein biosynthesis genes [86]. Disrupting this signaling cascade, either by degrading AHLs or inhibiting CviR activation, effectively suppresses violacein production and QS-dependent virulence factors [86]. A reduction in violacein production by at least 50% (%VI ≥ 50%) while maintaining less than 40% growth inhibition (%GI < 40%) is considered indicative of effective QSI. In this study, silver nanoparticles (AgNPs) synthesized by both *Streptomyces* sp. KE4D and *B. safensis* KE4K AgNPs exhibited varying degrees of QSI activity. *Streptomyces* sp. KE4D medium 5294 AgNPs (Supplementary Figure S5; Table 7) demonstrated the highest reduction in violacein production at a relatively low concentration (140–160 µg/mL). In contrast, *B. safensis* KE4K medium 5294 AgNPs primarily exhibited bactericidal activity at higher concentrations (160–200 µg/mL). The *Streptomyces* sp. KE4D medium 5333 AgNPs (Supplementary Figure S5; Table 7) did not show effective QSI, whilst *B. safensis* KE4K medium 5333 AgNPs displayed effective QSI at 160 µg/mL (33.33% GI; 52.02% VI). Previous studies suggest that the bacterial strain used for biosynthesis can significantly influence both antimicrobial and QSI activity. For example, Abudoleh and Mahasneh [87] identified 59 endophytes from berry surfaces in Ajloun, Jordan, of which 11 exhibited both QSI and antibacterial activity, while 42 displayed only antibacterial effects. Similarly, Qais et al. [88] reported that their *Streptomyces*-synthesized AgNPs could reduce violacein production by up to 80.34% at 4 µg/mL, though their study did not assess growth inhibition. An ideal QS inhibitor should suppress bacterial virulence without exerting bactericidal pressure, thereby minimizing the risk of resistance development [89]. Notably, *Bacillus* species are known to produce the quorum-quenching enzyme AiiA, which degrades acyl-homoserine lactone signals and reduces pathogenic virulence [90,91,92]. The observed QSI activity of *B. safensis* KE4K AgNPs may be attributed to AiiA acting as a capping agent during biosynthesis. Additionally, AgNPs themselves may contribute to QSI through direct interaction with CviR homologs, competitive inhibition of AHL binding, or oxidative stress-mediated disruption of QS signaling pathways [93]. Although these AgNPs demonstrated strong bactericidal activity against *C. violaceum,* optimizing their concentration could help shift the activity towards enhanced quorum quenching rather than bacterial killing (Table 7). Future studies will focus on elucidating the mechanism of action of these AgNPs by employing in silico molecular docking to predict their interactions at the molecular level.

AgNPs synthesized using *Streptomyces* sp. KE4D and *Bacillus safensis* KE4K exhibited significant, dose-dependent cytotoxicity against both prostate (DU-145) and breast (MCF-7) cancer cell lines. This effect is likely due to bioactive agents such as decanoic acid in the CFS used for AgNP functionalization. Decanoic acid, a medium-chain fatty acid with well-documented anti-proliferative properties, enhances the cytotoxicity of AgNPs by promoting their interaction with cancer cell membranes [94]. Consequently, incorporating decanoic acid as a capping agent represents a promising, synergistic approach to cancer therapy. This observation aligns with findings by Millaty et al. [95], who attributed the anti-tumor effects of *Aquilaria malaccensis* extracts to decanoic acid, amongst other metabolites. The *Streptomyces* sp. KE4D AgNPs displayed particularly low IC_50_ values against MCF-7 cells, possibly due to the high decanoic acid content in the medium Mannitol and medium 5294 AgNPs. This is in line with Balaji et al. [96], who reported hexadecanoic acid-mediated inhibition of MCF-7 cell growth. In contrast, while Samuel et al. [52] reported an IC_50_ of 50 µg/mL for *B. amyloliquefaciens*-derived AgNPs, the *B. safensis* KE4K AgNPs in this study achieved IC_50_ values below 3.5 µg/mL (Table 8). Although the present study did not include a normal cell line for comparison, Wypij et al. [97] investigated the cytotoxic effects of biosynthesized AgNPs from filamentous actinobacteria on both MCF-7 cancer cells and MRC-5 normal fibroblast cells using an MTT assay. Their findings revealed that the AgNPs exhibited significant cytotoxicity against MCF-7 cells, with an IC_50_ value of 12.9 μg/mL, while demonstrating markedly lower toxicity toward MRC-5 cells. While similar studies on DU-145 prostate cancer cells and normal cells using bacterial-synthesized AgNPs are currently lacking, the selective cytotoxicity observed in MCF-7 cells suggests a potential broader applicability of these AgNPs across different cancer types. These results highlight the superior anti-cancer potential of the synthesis method used and highlight the importance of selecting appropriate fermentation media and bacterial isolates to optimize the production of bioactive secondary metabolites for AgNP synthesis.

Furthermore, whereas most studies have focused on phyto-synthesized AgNPs for treating prostate cancer (DU-145) cell lines, our work offers a novel perspective by investigating AgNPs synthesized from *B. safensis* KE4K AgNPs and *Streptomyces* sp. KE4D endophytes. The lower IC_50_ values observed in this study compared to those reported for phyto-synthesized AgNPs [98,99,100] suggest that bacterial synthesis may enhance anti-cancer efficacy. These results underscore the need for continued research into microbial NP synthesis for therapeutic applications. Additionally, the positively charged *B. safensis* KE4K medium Mannitol AgNPs may benefit from improved cellular uptake and retention in tumor cells due to electrostatic attraction [101,102], further contributing to their potent cytotoxic effects.

## 4. Materials and Methods

### 4.1. Isolation of Endophytic Bacteria

Ten endophytes isolated from the healthy leaves of *Teclea nobilis,* collected from the Kakamega Tropical Rainforest in Kenya, which stretches from 0°10′ to 0°21′ N and longitude 34°44′ to 34°58′ E and an altitude of 1524 m above sea level [103], had been previously tested for their antimicrobial, QSI, and anti-biofilm potential ([104] Based on preliminary screening, two isolates (KE4D and KE4K; Figure 1A,B), which demonstrated antimicrobial activity and QSI activity, were selected for AgNP synthesis and bioactivity screening. The isolated endophytes were subcultured onto International *Streptomyces* Project 2 (ISP_2_) agar plates [105] to confirm the presence of pure cultures.

### 4.2. 16S rRNA Gene Amplification and Sequencing

Isolates KE4D and KE4K were cultured in 5 mL ISP_2_ broth at 30 °C. Bacterial DNA was isolated from 24–48 h cultures using the Quick-DNA^TM^ Fungal/Bacterial Miniprep kit (Zymo Research, Irvine, CA, USA). The 16S ribosomal RNA genes (1.5 kb) from isolates KE4D and KE4K were amplified using the universal primer sets F1 5′-AGAGTTTGATCITGGCTCAG-3-3′, R5 5′-ACGGITACCTTGTTACGACTT-3′ [106] and 27F-DEG 5′-AGAGTTTGATCMTGGCTCAG-3′, and 1492R-deg 5′-GGYTACCTTGTTACGACTT-3′ [107]. Amplification was performed in the MJ MINI^TM^ personal thermal cycler (Bio-Rad, Hercules, CA, USA) using the following PCR reaction conditions: initial DNA denaturation at 94 °C for 3 min, followed by 35 cycles of denaturation at 94 °C for 30 s, primer annealing at 52 °C for 1 min, and extension at 72 °C for 1 min and a final extension at 72 °C for 8 min. PCR products were purified and then sequenced using Sanger sequencing. Sequence data were processed using DNAMAN (version 7.0; Lynnon Corporation, Quebec, Canada) and subjected to identification using the NCBI-BLAST nucleotide database.

### 4.3. Bacterial Fermentations and Extract Characterization

Isolates were pre-cultured in 5 mL ISP_2_ broth for 2 d at 30 °C with shaking at 200 rpm. Isolates were then cultured in three different fermentation media (100 mL of medium Mannitol, medium 5294, and medium 5333). The composition of the fermentation media was as follows: medium Mannitol contained 20 g/L mannitol, 10 g/L yeast extract, and 1 g/L CaCO_3_; medium 5924 was composed of 10 g/L soluble starch, 2 g/L yeast extract, 10 g/L glucose, 10 g/L glycerol, 2.5 g/L corn steep liquor, 2 g/L peptone, 1 g/L NaCl, and 3 g/L CaCO_3_; and medium 5333 included 4 g/L yeast extract, 15 g/L soluble starch, 1 g/L K_2_HPO_4_, and 0.5 g/L MgSO_4_·7H_2_O [105]. Fermentations were carried out with agitation at 150 rpm for 5 d at 30 °C to enhance the production of secondary metabolites. Following incubation, the fermented cultures were centrifuged at 10,000 rpm for 10 min to obtain CFS. The CFS was then stored at 4 °C.

### 4.4. Ethyl Acetate Extraction, Fourier-Transform Infrared Spectroscopy and Gas Chromatography–Mass Spectrometry of Crude Extracts

*Streptomyces* sp. KE4D and *Bacillus safensis* KE4K were cultured in 5 mL of ISP_2_ broth for 2 d at 30 °C with shaking at 200 rpm. Thereafter, cultures were inoculated into 100 mL of medium Mannitol, medium 5924, and medium 5333 [105] in 250 mL glass flasks and incubated with shaking at 150 rpm at 30 °C for 5 d. Samples were then centrifuged at 10,000 rpm for 10 min, and CFS was collected. An equal volume of ethyl acetate (1:1) was added to each supernatant and then agitated at 30 °C for 1 h. The organic ethyl acetate layer was collected, and the remaining aqueous layer was subjected to a second extraction of 1:1 volume ethyl acetate, with agitation at 30 °C overnight. The ethyl acetate layer was again collected, and the combined organic layers were completely evaporated in a rotary evaporator (ILMVAC RObath, IKA, Staufen im Breisgau, Germany) at 40 °C. The dry extract was then resuspended in 4 mL methanol and added to a pre-weighed vial, after which the methanol was allowed to evaporate [108].

Fourier transmission infrared spectroscopy (FTIR) was undertaken to identify functional groups in the crude extracts. Dried crude bacterial extracts (2 mg) were mixed with 200 mg KBr and analyzed using the Bruker Alpha II Infrared Spectrometer (Billerica, MA, USA). Data were collected using ATR Diamond-1 Bounce (Bruker) with 24 sampling and background scans conducted in the range of 4000–400 cm^−1^. Data processing for smoothness, baseline correctness, and label peaks was conducted with Opus Spectroscopy software (OPUS 8.1 Build:8,1,29 (20180416; Bruker Optik GmbH; Ettlingen, Germany). Functional groups of the crude biosurfactants were identified using IR spectrum tables provided by Sigma-Aldrich (Darmstadt, Germany).

The volatile components from extracts were analyzed using gas chromatography–mass spectrometry (GC-MS; Shimadzu GCMS QP2010-SE with a Zebron ZB-5MSplus column 0.25 × 30 m (length); Columbia, MD, USA) × 0.25 μm (df). To eliminate interfering compounds, GC-MS analysis of sterile fermentation media alone was also performed and compared to the chromatograms of bacterial extracts. The components were identified by matching their recorded mass spectra with the standard mass spectra from the National Institute of Standards and Technology libraries’ data provided by the GC-MS system software (NIST11.LIB, version 2.0g), literature data, and standards of the main components.

### 4.5. Bacterial Silver Nanoparticle Synthesis

For AgNP biosynthesis, 10 mM silver nitrate (AgNO_3_; Sigma-Aldrich, Darmstadt, Germany) solution (precursor) was added to the CFS (secondary metabolites secreted by endophytic bacteria served as reducing agents) in a 1:1 ratio and incubated at 30 °C for 24 h under dark conditions with shaking at 150 rpm. The reduction of AgNO_3_ to elemental silver (Ag^+^) was observed by a change in color of the reaction mix from colorless to grey-black. As controls, the CFS was incubated without the addition of AgNO_3_, as well as respective sterile media together with AgNO_3_. The resulting AgNPs were centrifuged, and pellets were washed three times with sterile deionized water. Silver nanoparticles were standardized to a 10 mg/mL stock in sterile deionized water and stored at −20 °C for further investigation.

### 4.6. Characterization of Silver Nanoparticles

#### 4.6.1. UV-Visible Spectroscopy 

To confirm the presence of AgNPs, UV-visible spectroscopy of the biosynthesized AgNPs was carried out using the NanoPhotometer^®^ NP80 (Implemen, Munich, Germany) in nanovolume mode within a 200–800 nm range [109]. Deionized water was used as a blank for baseline correction.

#### 4.6.2. Fourier-Transform Infrared Spectroscopy

Fourier-transform infrared spectroscopy (FTIR) is useful for the identification of biomolecules involved in the capping and stabilization of AgNPs [110]. To understand and identify the surface chemistry of AgNPs, FTIR was performed using the Agilent Cary 630 spectrometer installed with Agilent MicroLab PC 5.1.22 to collect the data (Agilent, Santa Clara, CA, USA). ResolutionPro 5.0.0.395 was used to process the data for peaks and smoothing. The data were collected using ATR Diamond-1 Bounce with 30 background scans and 30 sampling scans in the range of 4000–650 cm^−1^ with a resolution of 4 cm^−1^ [109].

#### 4.6.3. High-Resolution Transmission Electron Microscopy (HR-TEM)

To examine size, shape, and distribution of the resulting AgNPs, HR-TEM was carried out using the JEOL 2100 microscope (JEOL Ltd., Tokyo, Japan) operating at 100 kV [111]. For sample viewing, 2 μL of each of the AgNP solutions was placed onto a carbon-coated copper grid and allowed to air dry. Size distribution analysis of the acquired TEM images was undertaken using the MIPAR–Image Analysis Software (version 3.3.4 for Windows PC).

#### 4.6.4. Energy-Dispersive X-Ray Analysis (EDX)

The elemental composition of the air-dried, carbon-coated samples was examined using an energy-dispersive attachment on a transmission electron microscope (JEOL 2100 HRTEM; JEOL Ltd., Tokyo, Japan) using the following instrumental conditions: accelerating voltage of 15 keV and counting time of 100 s [112]. The dispersal of X-rays from the AgNPs was detected after exposing the AgNPs to a high concentration of electrons.

#### 4.6.5. Zeta Potential Assessment

Zeta potential analysis was carried out using the Zetasizer Nano ZS90 (Malvern Panalytical, Malvern, UK; equipped with software version 7.10). The AgNPs were diluted 10-fold with distilled water, and 100 μL aliquots were then sampled in dynamic light scattering (DLS) cuvettes and analyzed for equivalent surface charge using zeta potential criteria of Honary and Zahir [41].

### 4.7. Biological Activity Assessment of Endophytic Bacteria AgNPs

#### 4.7.1. Antimicrobial Activity Assessment

Antimicrobial susceptibility testing of the biosynthesized AgNPs was carried out using the agar well diffusion assay. Three Gram-positive (vancomycin-resistant *Enterococcus faecalis* ATCC 51299, *Listeria monocytogenes* ATCC 19111, methicillin-resistant *Staphylococcus aureus* (MRSA) ATCC 43300) and three Gram-negative (*Acinetobacter baumannii* ATCC 19606, β-lactam-resistant *Escherichia coli* ATCC 35218, and multi-drug resistant *Pseudomonas aeruginosa* ATCC 27853) bacteria were grown at 37 °C overnight on Mueller–Hinton (MH) agar plates, with the exception of *L. monocytogenes*, which was grown overnight on Mueller–Hinton fastidious agar (Becton Dickinson, Franklin Lakes, NJ, USA). Inocula equivalent to a 0.5 McFarland standard were prepared and used to swab the surface of MH plates. A sterile 4 mm wide cork borer was used to punch wells into the agar. Four hundred micrograms (40 μL of a 10 mg/mL stock solution) of AgNPs were then loaded into each well [113]. Agar plates were then incubated for 24 h at 37 °C. Ampicillin (AMP10), gentamicin (CN10), and tetracycline (TE30) discs were used as the antibiotic controls [113]. Following incubation, zone diameters ≥ 16 mm were regarded as being strong, 11–15 mm as intermediate, and ≤10 mm as weak antimicrobial activity [113].

#### 4.7.2. Anti-Quorum Sensing Activity

The QSI activity of the biosynthesized AgNPs was quantified using the violacein inhibition assay with *Chromobacterium violaceum* ATCC 12472 as the bio-indicator organism [113]. The long-chain acyl-homoserine lactone (AHL)-producing *C. violaceum* biosensor [113] was cultured in 3 mL of Lysogeny Broth (LB) at 30 °C with the addition of increasing concentrations of respective AgNPs (0; 100; 120; 140; 160; 180; 200 μg/mL). Vanillin (50–800 µg/mL; Sigma-Aldrich, Darmstadt, Germany) was used as the QSI-positive control [113].

For this assay, growth (OD_600 nm_) and violacein production (OD_560nm_) were determined following overnight incubation using the Glomax Multi+ Detection System (Promega, Madison, WI, USA). One ml of an overnight culture of *C. violaceum* was centrifuged at 10,000 rpm for 10 min to precipitate insoluble violacein. The culture supernatant was discarded, and the pellet homogenously resuspended in 1 mL of dimethyl sulfoxide (DMSO) [113]. The solution was then centrifuged again at 10,000 rpm for 10 min to remove the cells, and the violacein was quantified at OD_560_ nm [113]. The %violacein inhibition (%VI) was calculated as follows [113]:

$$\%Violacein\;inhibition=(\frac{\mathrm{control}\;\mathrm{OD}560\;\mathrm{nm}-\mathrm{test}\;\mathrm{OD}560\;\mathrm{nm}}{\mathrm{control}\;\mathrm{OD}560\;\mathrm{nm}})\times 100$$

AgNP samples at any given concentration that exhibited a percentage growth inhibition (%GI) ≥ 40% relative to the untreated control were considered bactericidal rather than inhibiting QS. AgNP samples that exhibited %VI ≥ 50% with the %GI < 40% were considered good QS inhibitors.

#### 4.7.3. Anti-Cancer Assays

##### Cell Culture

The human breast cancer cell line MCF-7 (ATCC HTB-22^TM^; RRID:CVCL-0031) and the human prostate cancer cell line DU-145 (ATCC HTB-81^TM^; RRID:CVCL_0105) were purchased from the American Type Culture Collection (ATCC, Manassas, VA, USA) and cultured in Eagle’s Minimal Essential Medium (EMEM, Sigma-Aldrich; Darmstadt, Germany) supplemented with 10% fetal bovine serum (FBS) and 1% penicillin/streptomycin (Sigma-Aldrich, Darmstadt, Germany). Both cell lines were incubated at 37 °C in a humidified atmosphere of 5% CO_2_ and were routinely grown in 25 cm^2^ culture flasks. After reaching 70% confluence, cells were trypsinized (0.25% Trypsin–EDTA in Hank’s balanced salt solution), followed by centrifugation (1300× *g* for 7 min) and re-suspended in culture medium for the assay [114].

##### Cytotoxicity Test—MTT Assay

The DU-145 and MCF-7 cell lines were used to assess the effects of biosynthesized AgNPs on prostate and breast cancer cells, respectively. To evaluate cell viability and metabolic activity, the MTT (3-[4,5-dimethylthiazol-2-yl]-2,5-diphenyl tetrazolium bromide) colorimetric assay was performed [115]. Briefly, the MCF-7 and DU-145 cells at a seeding density of 1 × 10^4^ were added to each well of a 96-well flat-bottom plate and allowed to adhere for 24 h at 37 °C in a humidified 5% CO_2_ atmosphere. After 24 h, stock solutions of each biosynthesized AgNP were serially diluted from 1250 µg/mL to 2.44 µg/mL. The positive control included a 1 mg/mL stock of 5-fluorouracil (Sigma-Aldrich), a known cytotoxic chemotherapeutic agent at 10, 25, 50, and 100 µg/mL concentrations. Cells were then re-incubated for 24 h at 37 °C in 5% CO_2_ chamber. Following incubation, the cells were observed using bright-field microscopy (20× magnification; EVOS^®^ FL Cell Imaging System, Thermo Fisher Scientific, Waltham, MA, USA) for morphological changes. Thereafter, 10 μL of MTT (5 mg/mL) was added to each well, and the plate was incubated further. After 4 h, the supernatant in each well was carefully removed, and 100 μL of DMSO was added to dissolve the resulting formazan crystals, the amount of which could be quantified by determining absorbance at 570 nm and 750 nm using the Glomax Multi+ Detection System (Promega, Madison, WI, USA). The cytotoxic effects of the AgNPs were calculated using the following formula [116]:

$$\%Cell\;viability=\frac{\mathrm{Test}\;\mathrm{Avg}.\;\left(\mathrm{OD}560\;\mathrm{nm}\;-\;\mathrm{OD}750\;\mathrm{nm}\right)-\mathrm{Blank}\;\mathrm{Avg}.\;\left(\mathrm{OD}560\;\mathrm{nm}-\mathrm{OD}750\;\mathrm{nm}\right)}{\mathrm{Untreated}\;\mathrm{Avg}.\;\left(\mathrm{OD}560\;\mathrm{nm}-\mathrm{OD}750\;\mathrm{nm}\right)-\mathrm{Blank}\;\mathrm{Avg}.\;\left(\mathrm{OD}560\;\mathrm{nm}-\mathrm{OD}750\;\mathrm{nm}\right)}\times 100$$

Thereafter, the IC_50_ value (inhibitory concentration; a concentration of a compound inhibiting 50% of the cell growth) was calculated using GraphPad Prism software (version 10, Dotmatics, Boston, MA, USA) and compared to that of the untreated control group.

### 4.8. Statistical Analysis

Data were statistically analyzed using GraphPad Prism software (version 10; GraphPad Software Inc., Boston, MA, USA). Data are presented as a mean ± SD (*n* = 3). The difference in violacein inhibition mean values between biosynthesized AgNPs was determined using repeated-measures ANOVA, with *p ≤* 0.05 considered significant using Tukey’s post hoc test. Viability results were reported as an average ± SD of three replicate values. The IC_50_ values, *p*-values, and graphics were also calculated using the dose–response model on the GraphPad Prism software.

## 5. Conclusions

The synthesis of AgNPs using *Streptomyces* sp. KE4D and *B. safensis* KE4K endophytes from *T. nobilis* demonstrates significant potential in antimicrobial and anti-cancer applications, particularly by promoting the sustainability of bioprospecting microorganisms rather than relying on whole plant species from the Kakamega rainforest. The choice of fermentation medium plays a critical role, as it directly influences the yield, morphology, and bioactivity of the AgNPs. Starch-rich media, such as medium Mannitol and medium 5294, resulted in AgNPs with enhanced bioactive properties, likely due to the interactions between compounds produced by *Streptomyces* sp. KE4D and *B. safensis* KE4K during fermentation. This underscores the importance of optimizing culture conditions while considering the bacterial source and its metabolic behavior. Functionalizing natural compounds with AgNPs further boosts their bioactivity and application longevity. The findings from this study suggest that AgNPs synthesized from microorganisms isolated from medicinal plants like *T. nobilis* could serve as promising candidates for next-generation antimicrobial and anti-cancer therapies. Future research should explore the underlying mechanisms of these interactions and investigate the potential for integrating AgNPs with existing treatments to combat drug resistance and cancer, thus advancing sustainable therapeutic development from natural sources.

## Figures and Tables

**Table 1:** Comparison of the functional groups present in the *Streptomyces* sp. KE4D and *Bacillus safensis* KE4K crude bacterial extracts obtained following fermentation in medium Mannitol, medium 5294, and medium 5333 and the respective biosynthesized silver nanoparticles (AgNPs) using Fourier-transform infrared spectroscopy.

Crude Extract Samples	Functional Groups
KE4D	
Mannitol	Hydroxyl (O-H), carbon dioxide (O=C=O), bonded stretching of amines/amides (N-H/C-H/O-H), alkene (C=C), isothiocyanate (N=C=S), alkyl halides (R-X).
5294	Hydroxyl (O-H), carbon dioxide (O=C=O), alkane (C-H), carboxylic acid (R-COOH), alkene (C=C), isothiocyanate (N=C=S), alkyl halides( R-X).
5333	Hydroxyl (O-H), carbon dioxide (O=C=O), alkane (C-H), cyanide (C-N), alkene (C=C), isothiocyanate (N=C=S), alkyl halides(R-X).
KE4K	
Mannitol	Hydroxyl (O-H), carbon dioxide (O=C=O), alkane (C-H), alkene (C=C), isothiocyanate (N=C=S), alkyl amine (R-NH_2_).
5294	Hydroxyl (O-H), carbon dioxide (O=C=O), alkene (C=C), nitrile (CΞN), aromatic compound (C-H), alkyl amine (R-NH_2_).
5333	Hydroxyl (O-H), carbon dioxide (O=C=O), alkene (C=C), nitrile (CΞN), aromatic compound (C-H), alkyl amine (R-NH_2_).
**AgNPs**	**Functional groups**
KE4D	
Mannitol	Hydroxyl (O-H), carbon dioxide (O=C=O), alkyne (CΞC), isothiocyanate (N=C=S), nitro- (N-O), alkane (C-H), alcohol (C-O).
5294	Hydroxyl (O-H), alkane (C-H), carbon dioxide (O=C=O), alkyne (CΞC), isothiocyanate (N=C=S), alkene (C=C), nitro- (N-O), alkyl aryl ether (C-O), sulfoxide (S=O).
5333	Amine (N-H), carbon dioxide (O=C=O), alkyne (CΞC), isothiocyanate (N=C=S), nitro- (N-O), fluoro-compound (C-F), sulfonamide (S=O), aromatic amine (C-N), phenol (O-H), trisubstituted alkene (C=C).
KE4K	
Mannitol	Hydroxyl (O-H), amine salt (N-H), carbon dioxide (O=C=O), isothiocyanate (N=C=S), nitro- (N-O), ether (C-O-C).
5294	Hydroxyl (O-H), amine salt (N-H), carbon dioxide (O=C=O), alkyne (CΞC), aromatic compound (C-H), alkene (C=C), sulfonamide (S=O).
5333	Hydroxyl (O-H), carbon dioxide (O=C=O), nitrile (CΞN), aromatic compound (C-H), sulfoxide (S=O).
	
**Fermentation media control**	
Mannitol	Hydroxyl (O-H), carbon dioxide (O=C=O), alkane (C-H), alkene (C=C), carboxylic acid (R-COOH), ester (R-COO-R).
5294	Hydroxyl (O-H), carbon dioxide (O=C=O), ester (R-COO-R), carboxylic acid (R-COOH), nitrile (CΞN), ketone RC(=O)R.
5333	Hydroxyl (O-H), carbon dioxide (O=C=O), ester (R-COO-R), sulfoxide (S=O), carboxylic acid (R-COOH), ketone RC(=O)R.

**Table 2:** GC-MS analysis of the crude medium Mannitol fermentation extracts of endophytic *Streptomyces* sp. KE4D and *Bacillus safensis* KE4K and medium Mannitol fermentation control broth extract.

Compound	Medium Mannitol Control		KE4D Mannitol		KE4K Mannitol	
	Rt (min)	%Area	Rt (min)	%Area	Rt (min)	%Area
1,2-Benzenedicarboxylic acid, bis(2-methylpropyl	16.614	57.04	ND	ND	ND	ND
2,5-Hexanedione, 3,4-dihydroxy-3,4-dimethyl-	ND	ND	ND	ND	6.347	18.36
2,5-Hexanedione, 3,4-dihydroxy-3,4-dimethyl-	ND	ND	ND	ND	7.973	1.26
Acetamide, N-(2-methylpropyl)-	ND	ND	ND	ND	6.413	6.41
cis-10-Heptadecenoic acid	23.272	4.87	ND	ND	ND	ND
Cyclopropaneacetic acid, 2-hexyl-	ND	ND	12.484	2.09	ND	ND
Dodecanoic acid, isooctyl ester	21.65	2.42	ND	ND	ND	ND
Eicosanoic acid	ND	ND	20.418	3.17	ND	ND
Hexadecanoic acid	ND	ND	18.728	7.83	ND	ND
Hexadecanoic acid, methyl ester	ND	ND	17.151	1.69	ND	ND
Hexanedioic acid, bis(2-methylpropyl) ester	14.392	6.84	ND	ND	ND	ND
Isovaline, 3-hydroxy-	ND	ND	ND	ND	6.347	18.36
Isovaline, 3-hydroxy-	ND	ND	ND	ND	6.413	6.41
Malic Acid	ND	ND	ND	ND	7.244	4.23
N(1),N(1)-Diethyl-1,2-butanediamine	ND	ND	ND	ND	6.478	4.03
N-(3-Methylbutyl)acetamide	ND	ND	ND	ND	7.705	6.61
Pentadecanoic acid	ND	ND	16.337	2.14	ND	ND
Pentadecanoic acid	ND	ND	16.49	11.86	ND	ND
Pentadecanoic acid, 14-methyl-, methyl ester	ND	ND	17.796	1.27	ND	ND
Pyrazine, tetramethyl-	ND	ND	ND	ND	8.017	0.47
Pyrrolo [1,2-a]pyrazine-1,4-dione, hexahydro-3-(2	ND	ND	20.21	35.08	ND	ND
Tetradecanoic acid	ND	ND	15.11	0.92	ND	ND
Tetradecanoic acid, 5,9,13-trimethyl-, methyl ester	ND	ND	19.301	2.13	ND	ND
Tridecanoic acid, 12-methyl-, methyl ester	ND	ND	15.813	6.01	ND	ND

ND = Not detected in the chromatogram. Rt = Retention time in minutes.

**Table 3:** GC-MS analysis of the crude medium 5294 fermentation extracts of endophytic *Streptomyces* sp. KE4D and *Bacillus safensis* KE4K and medium 5294 fermentation control broth extract.

Compound	Medium 5294 Control		KE4D 5294		KE4K 5294	
	Rt (min)	%Area	Rt (min)	%Area	Rt (min)	%Area
1,2-Benzenedicarboxylic acid, bis(2-methylpropyl) ester	16.838	66.45	ND	ND	ND	ND
1-Methyl-2-morpholin-4-ylethyl acetate	ND	ND	ND	ND	13.68	3.55
1-Penten-3-one, 1-(2,6,6-trimethyl-1-cyclohexen-1-yl)	ND	ND	13.996	1.84	ND	ND
2,4-Imidazolidinedione, 5-(2-methylpropyl)-, (S)-	ND	ND	13.552	5.3	ND	ND
3,7-Cyclodecadiene-1-methanol,. alpha.,.alpha.,4,8 tetramethyl	ND	ND	14.05	1.22	ND	ND
4-Methyloctanoic acid	ND	ND	9.787	3.01	ND	ND
6-Octadecenoic acid, (Z)-	23.717	2.51	ND	ND	ND	ND
Butanoic acid, 3-methyl-	ND	ND	ND	ND	5.375	23.59
Dibutyl phthalate	ND	ND	17.959	8.33	17.964	2.26
Dioxane-2,5-dimethanol	ND	ND	8.977	2.17	ND	ND
Dodecanoic acid, isooctyl ester	22.059	2.85	ND	ND	ND	ND
Eicosane	ND	ND	18.713	2.25	ND	ND
Eicosanoic acid	ND	ND	19.658	2.32	ND	ND
Glutaric acid, di(isobutyl) ester	13.472	2.4	ND	ND	ND	ND
Heptadecane, 2,6,10,15-tetramethyl-	ND	ND	13.47	3.62	ND	ND
Hexanedioic acid, bis(2-methylpropyl) ester	14.529	11.29	ND	ND	ND	ND
i-Propyl 12-methyltetradecanoate	ND	ND	16.073	6.08	ND	ND
Isobutyl isothiocyanate	ND	ND	ND	ND	6.482	2.17
-Isopropyl-2,4-imidazolidinedione	ND	ND	12.553	1.72	ND	ND
l-(+)-Ascorbic acid 2,6-dihexadecanoate	ND	ND	18.103	3.96	ND	ND
N-Methyl-3-hydroxymethylpyrrolidin-2-one	ND	ND	ND	ND	13.773	3.5
Propanamide, N-methyl-	ND	ND	ND	ND	5.455	19.62
Propanol, 2,2-dimethyl-, acetate	ND	ND	3.818	1.32	ND	ND
Pyrazine, tetramethyl-	ND	ND	ND	ND	7.67	4.1
Pyrrolo [1,2-a]pyrazine-1,4-dione, hexahydro-3-(2-methylpropyl)	ND	ND	17.528	2	17.869	0.41
Pyrrolo [1,2-a]pyrazine-1,4-dione, hexahydro-3-(2-methylpropyl)	ND	ND	17.833	2.29	ND	ND
Tetradecanoic acid	ND	ND	11.38	1.64	ND	ND

ND = Not detected in chromatogram. Rt = Retention time in minutes.

**Table 4:** GC-MS analysis of the crude medium 5333 fermentation extracts of endophytic *Streptomyces* sp. KE4D and *Bacillus safensis* KE4K and the medium 5333 fermentation control broth extract.

Compound	Medium 5333 Control		KE4D 5333		KE4K 5333	
	Rt (min)	%Area	Rt (min)	%Area	Rt (min)	%Area
1,2-Benzenedicarboxylic acid, bis(2-methylpropyl)	16.878	42.95	ND	ND	ND	ND
2,4-Imidazolidinedione, 5-(2-methylpropyl)-, (S)-	13.881	15.59	ND	ND	ND	ND
2,4-Imidazolidinedione, 5-methyl-	11.658	4.88	ND	ND	ND	ND
5-Isopropyl-2,4-imidazolidinedione	12.853	3.1	ND	ND	ND	ND
5-n-Propylhydantoin	ND	ND	12.603	5.5	ND	ND
6,19-Cycloandrostane-3,7-diol, 3.beta.-methoxy-	ND	ND	ND	ND	29.23	4
Benzonitrile, 3-benzyloxy-	ND	ND	18.687	14.76	ND	ND
Dibutyl phthalate	ND	ND	18.077	15.54	18.087	3.48
Diisooctyl phthalate	ND	ND	ND	ND	29.388	17.17
Dodecane, 2,6,11-trimethyl-	ND	ND	ND	ND	11.464	1.48
Eicosane	ND	ND	14.474	0.73	15.602	1.97
Glutaric acid, isobutyl undecyl ester	13.51	1.34	ND	ND	ND	ND
Hexanedioic acid, bis(2-methylpropyl) ester	14.567	6.16	ND	ND	ND	ND
Phthalic acid, di(4,4-dimethylpent-2-yl) ester	ND	ND	ND	ND	29.31	4.87
Propanoic acid, 2-methyl-, 3-hydroxy-2,4,4-trimethylpentyl	11.419	1.5	ND	ND	ND	ND
Pyrrolo [1,2-a]pyrazine-1,4-dione, hexahydro-3-(2-methylpropyl)-	ND	ND	17.619	3.84	ND	ND
Pyrrolo [1,2-a]pyrazine-1,4-dione, hexahydro-3-(2-methylpropyl)-	ND	ND	17.935	2.54	ND	ND
Tricosane-2,4-dione	ND	ND	ND	ND	13.542	18.48

ND = Not detected in chromatogram. Rt = Retention time in minutes.

**Table 5:** Comparison of size, morphology, aggregation, and zeta potential of silver nanoparticles (AgNPs) synthesized from isolates *Streptomyces* sp. KE4D and *Bacillus safensis* KE4K following fermentation using different media.

Characteristic	Medium Mannitol		Medium 5294		Medium 5333	
	KE4D	KE4K	KE4D	KE4K	KE4D	KE4K
Size range	5–55 nm	5–55 nm	5–49 nm	4–55 nm	4–49 nm	4–49 nm
Morphology	Spherical	Spherical, triangular, rod-shaped	Spherical	Spherical	Spherical	Spherical
Aggregation	+++	+++	+	++	++	+++
ζ-potential (mV)	−17.0	+16.2	−17.0	−16.8	−17.1	−12.1

Aggregation criteria: + = weakly agglomerated, ++ = intermediately agglomerated, and +++ = highly agglomerated; ζ-potential (mV) criteria: −5 mV to +5 mV = low stability, fast aggregation, =20 mV = short term stability, >+30 mV = stable (strongly cationic), <−30 mV = stable (strongly anionic), ≥+60 mV = excellent stability, and ≤−60 mV = excellent stability [41].

**Table 6:** Antimicrobial activity of silver nanoparticles (AgNPs) biosynthesized from endophytic bacteria *Streptomyces* sp. KE4D and *Bacillus safensis* KE4K extracts against clinical isolates using the agar well diffusion assay.

AgNPs (400 μg)	Diameter of Inhibition Zone (mm)					
	*E. faecalis* ATCC 51299	*L. monocytogenes* ATCC 19111	*S. aureus* ATCC 43300	*A. baumannii* ATCC 19606	*E. coli* ATCC 35218	*P. aeruginosa* ATCC 27853
KE4D Mannitol	12	*6*	14	14	13	14
KE4D 5294	*10*	*0*	**16**	14	**16**	14
KE4D 5333	*7*	*5*	15	15	15	13
						
KE4K Mannitol	**17.5**	*10*	**16**	14.5	15	*6*
KE4K 5294	**15.5**	*0*	*0*	11.5	13.5	*0*
KE4K 5333	*0*	*0*	*0*	*8*	*9*	*0*
						
Ampicillin (AMP10)	17	10	12	0	15	0
Gentamicin (CN10)	11	0	13	0	12	18
Tetracycline (TET30)	0	15	16	15	9	0

Gram-positive bacteria: vancomycin-resistant *Enterococcus faecalis* ATCC 51299, *Listeria monocytogenes* ATCC 19111, and methicillin-resistant *Staphylococcus aureus* ATCC 43300. Gram-negative bacteria: *Acinetobacter baumannii* ATCC 19606, β-lactam-resistant *Escherichia coli* ATCC 35218, and multidrug-resistant *Pseudomonas aeruginosa* ATCC 27853. Strong antimicrobial activity (red): ≥16 mm. Intermediate antimicrobial activity (yellow/italics): 11–15 mm. Weak antimicrobial activity (green/bold): ≤10 mm.

**Table 7:** Percentage growth (%GI) and violacein inhibition (%VI) of silver nanoparticles (AgNPs) synthesized from endophytic bacteria *Streptomyces* sp. KE4D and *Bacillus safensis* KE4K extracts in the *Chromobacterium violaceum* ATCC 12472 inhibition assay.

AgNPs	100 µg/mL		120 µg/mL		140 µg/mL		160 µg/mL		180 µg/mL		200 µg/mL	
	%GI	%VI	%GI	%VI	%GI	%VI	%GI	%VI	%GI	%VI	%GI	%VI
KE4D Mannitol	3.54	37.08	11.36	41.90	11.69	44.56	**11.99**	**50.08**	**11.88**	**50.55**	**18.68**	**50.06**
KE4D 5294	8.04	18.19	8.71	21.48	**8.25**	**85.12**	*76.49*	*87.34*	*79.52*	*92.00*	*89.32*	*98.61*
KE4D 5333	4.64	14.93	−4.47	13.18	4.53	14.02	8.35	14.91	18.01	20.57	−4.51	20.80
												
KE4K Mannitol	6.05	25.38	11.80	33.25	**31.45**	**55.94**	*83.59*	*75.07*	*90.32*	*69.22*	*92.20*	*80.88*
KE4K 5294	14.65	15.98	21.21	26.01	**25.36**	**49.84**	*90.95*	*80.85*	*91.59*	*83.79*	*91.59*	*86.50*
KE4K 5333	26.61	42.25	28.67	43.74	28.11	47.26	**33.33**	**52.02**	*54.94*	*72.43*	*91.74*	*78.03*
	**50 µg/mL**		**100 µg/mL**		**200 µg/mL**		**400 µg/mL**		**800 µg/mL**			
	**%GI**	**%VI**	**%GI**	**%VI**	**%GI**	**%VI**	**%GI**	**%VI**	**%GI**	**%VI**		
Vanillin control	6.26	−2.76	2.77	14.48	8.70	36.57	12.03	56.98	17.88	68.29		

Growth was measured at OD 600 nm and violacein at OD 560 nm. Samples exhibiting ≥50% VI with <40% GI were considered good QSIs. AgNPs exhibiting ≥50% VI with <40% GI (good QSIs) are indicated in green/bold. AgNPs exhibiting ≥50% VI with ≥40% GI (strong antimicrobials) are indicated in red/italics.

**Table 8:** The IC_50_ values of silver nanoparticles (AgNPs) biosynthesized from endophytic bacteria *Streptomyces* sp. KE4D and *Bacillus safensis* KE4K extracts against MCF-7 breast and DU-145 prostate cancer cell lines.

AgNP Sample	MCF-7 IC_50_ Value (µg/mL)	DU-145 IC_50_ Value (µg/mL)
KE4D medium Mannitol	3.000	2.277
KE4D medium 5294	3.003	2.059
KE4D medium 5333	2.034	2.335
		
KE4K medium Mannitol	2.237	1.938
KE4K medium 5294	2.990	2.020
KE4K medium 5333	3.007	2.399
5-fluorouracil control	21.750	43.020

## Data Availability

The data that support the findings of this study are available from the corresponding author upon reasonable request.

## Supplementary Materials

The following supporting information can be downloaded at https://www.mdpi.com/article/10.3390/ijms26073306/s1.
