# Supplementary material for: Antimicrobial, Quorum Sensing Inhibition, and Anti-Cancer Activities of Silver Nanoparticles Synthesized from Kenyan Bacterial Endophytes of Teclea nobilis
Source: Int J Mol Sci. 2025 Apr 2;26(7):3306. doi: 10.3390/ijms26073306 (PMC11989699; doi:10.3390/ijms26073306)
Supplement: Supplementary file 1 [file ijms-26-03306-s001.zip › ijms-3512849-supplementary.pdf]

Antimicrobial, Quorum Sensing Inhibition, and Anti-Cancer Activities of Silver Nanoparticles Synthesized from Kenyan Bacterial Endophytes of *Teclea nobilis*

Farzana Mohamed and Hafizah Yousuf Chenia

Discipline: Microbiology (Westville Campus), School of Life Sciences, University of KwaZulu-Natal, Private Bag X54001, Durban, KwaZulu-Natal, South Africa, 4000

Supplementary Information

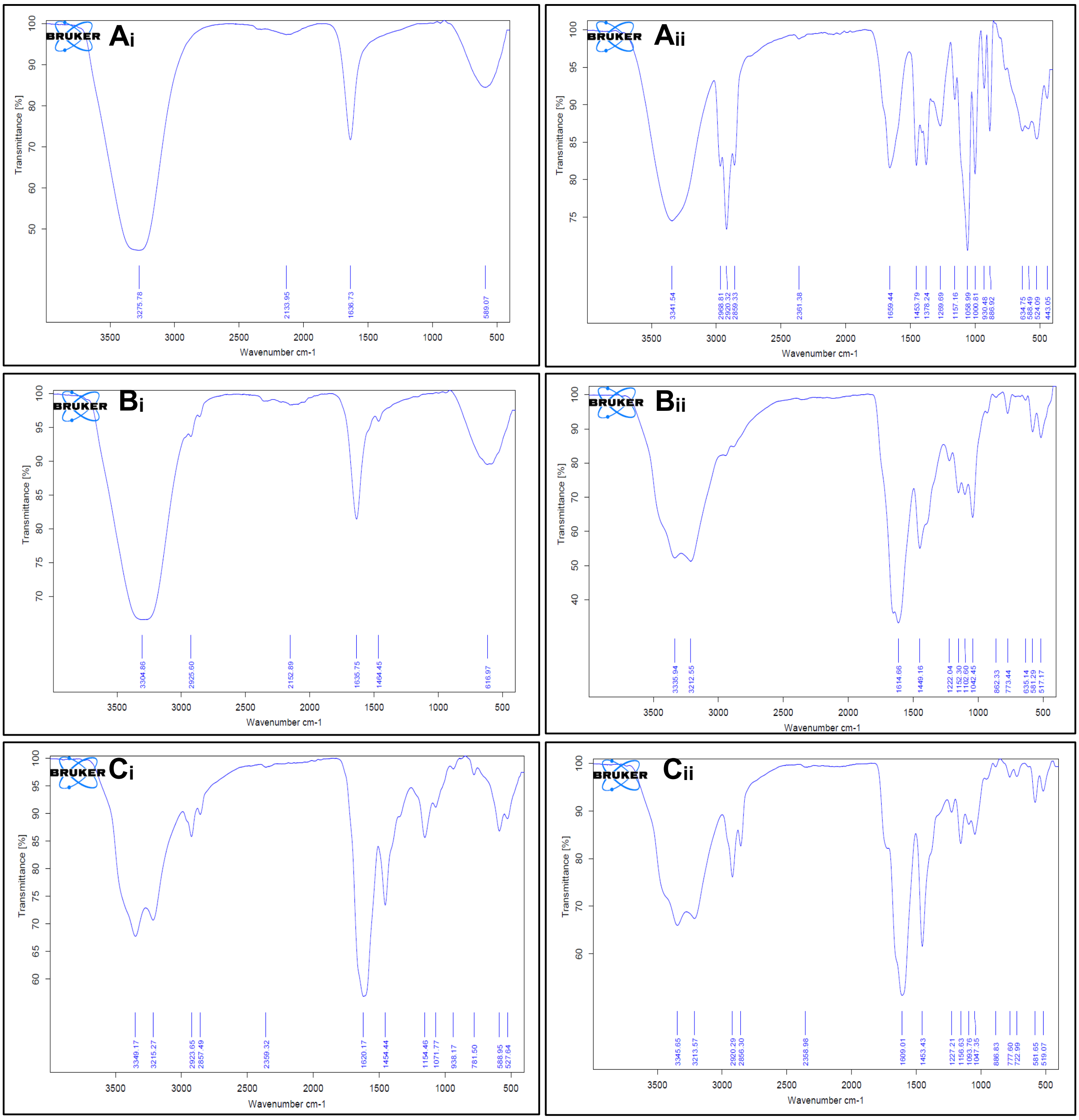

Figure S1. Fourier-transform infrared spectra of crude Kenyan endophytic bacterial extracts: (A_i) *Streptomyces* sp. KE4D medium Mannitol, (A_{ii}) *Bacillus safensis* KE4K medium Mannitol, (B_i) *Streptomyces* sp. KE4D medium 5294, (B_{ii}) *B. safensis* KE4K medium 5294, (C_i) *Streptomyces* sp. KE4D medium 5333, and (C_{ii}) *B. safensis* KE4K medium 5333

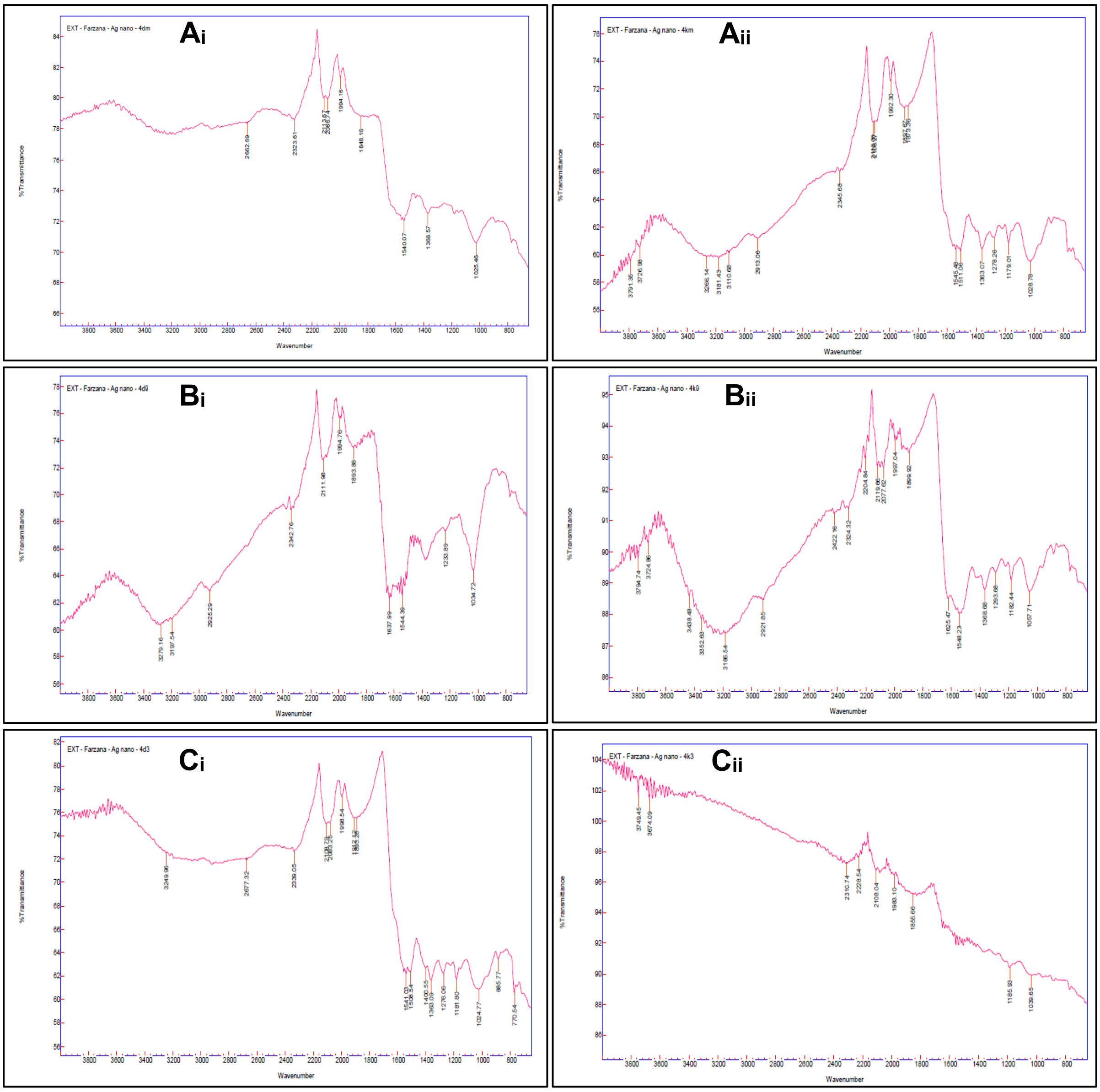

Figure S2. Fourier-transform infrared spectra of silver nanoparticles (AgNPs) biosynthesised from cell-free supernatants of two Kenyan bacterial endophytes: (A_i) *Streptomyces* sp. KE4D medium Mannitol, (A_{ii}) *Bacillus safensis* KE4K medium Mannitol, (B_i) *Streptomyces* sp. KE4D medium 5294, (B_{ii}) *B. safensis* KE4K medium 5294, (C_i) *Streptomyces* sp. KE4D medium 5333, and (C_{ii}) *B. safensis* KE4K medium 5333

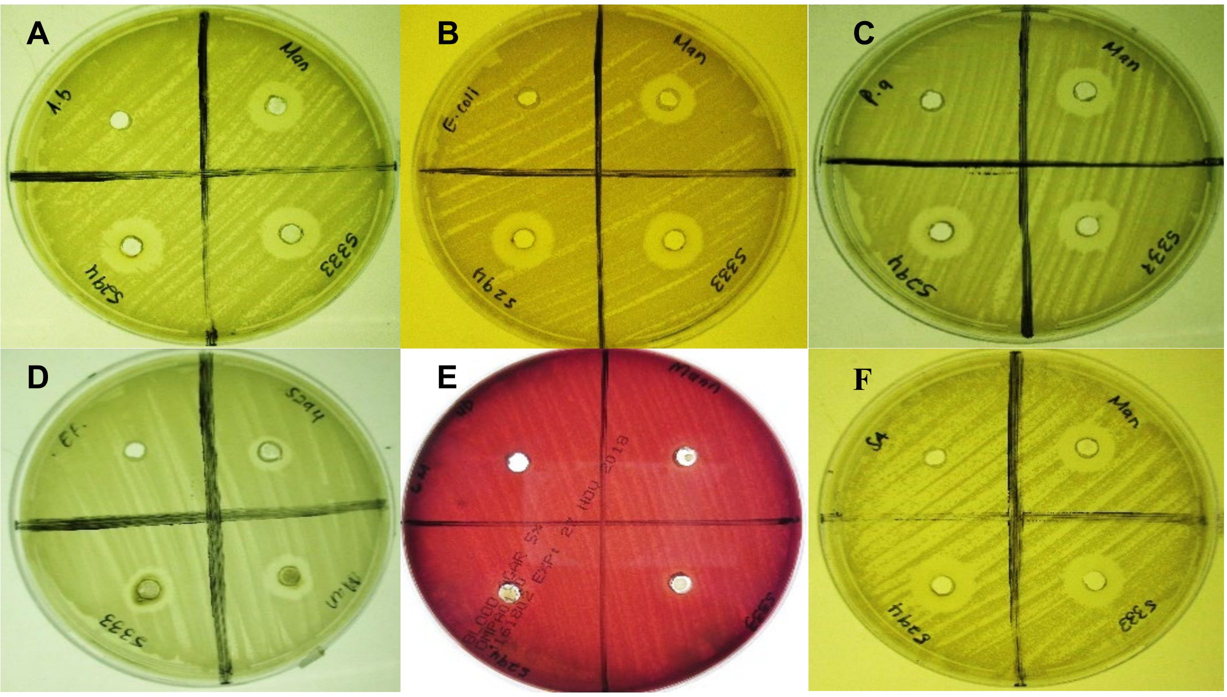

Figure S3. Antimicrobial effects of silver nanoparticles (AgNPs), biosynthesised from cell-free supernatants, of endophytic bacterium *Streptomyces* sp. KE4D against (A) *Acinetobacter baumannii* ATCC 19606, (B) *Escherichia coli* ATCC 35218, (C) *Pseudomonas aeruginosa* ATCC 27853, (D) *Enterococcus faecalis* ATCC 51299, (E) *Listeria monocytogenes* ATCC 19111 and (F) *Staphylococcus aureus* ATCC 43300

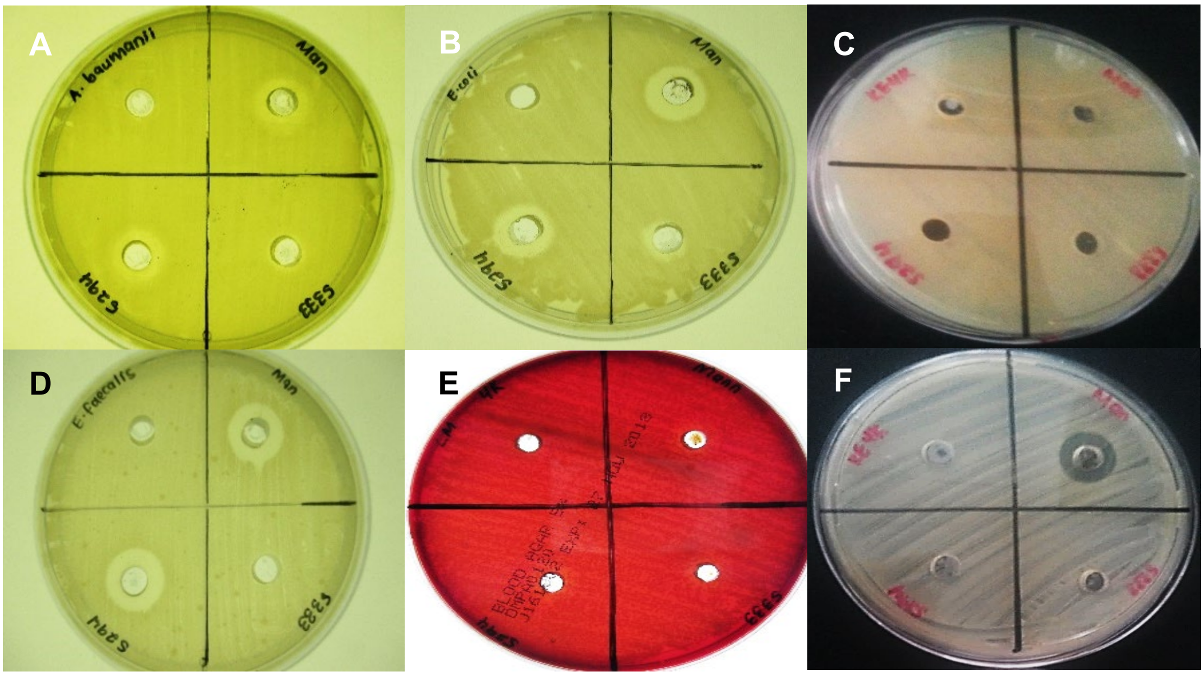

Figure S4. Antimicrobial effects of silver nanoparticles (AgNPs), biosynthesised from cell-free supernatants, of endophytic bacterium *Bacillus safensis* KE4K against (A) *Acinetobacter baumannii* ATCC 19606, (B) *Escherichia coli* ATCC 35218, (C) *Pseudomonas aeruginosa* ATCC 27853, (D) *Enterococcus faecalis* ATCC 51299, (E) *Listeria monocytogenes* ATCC 19111 and (F)

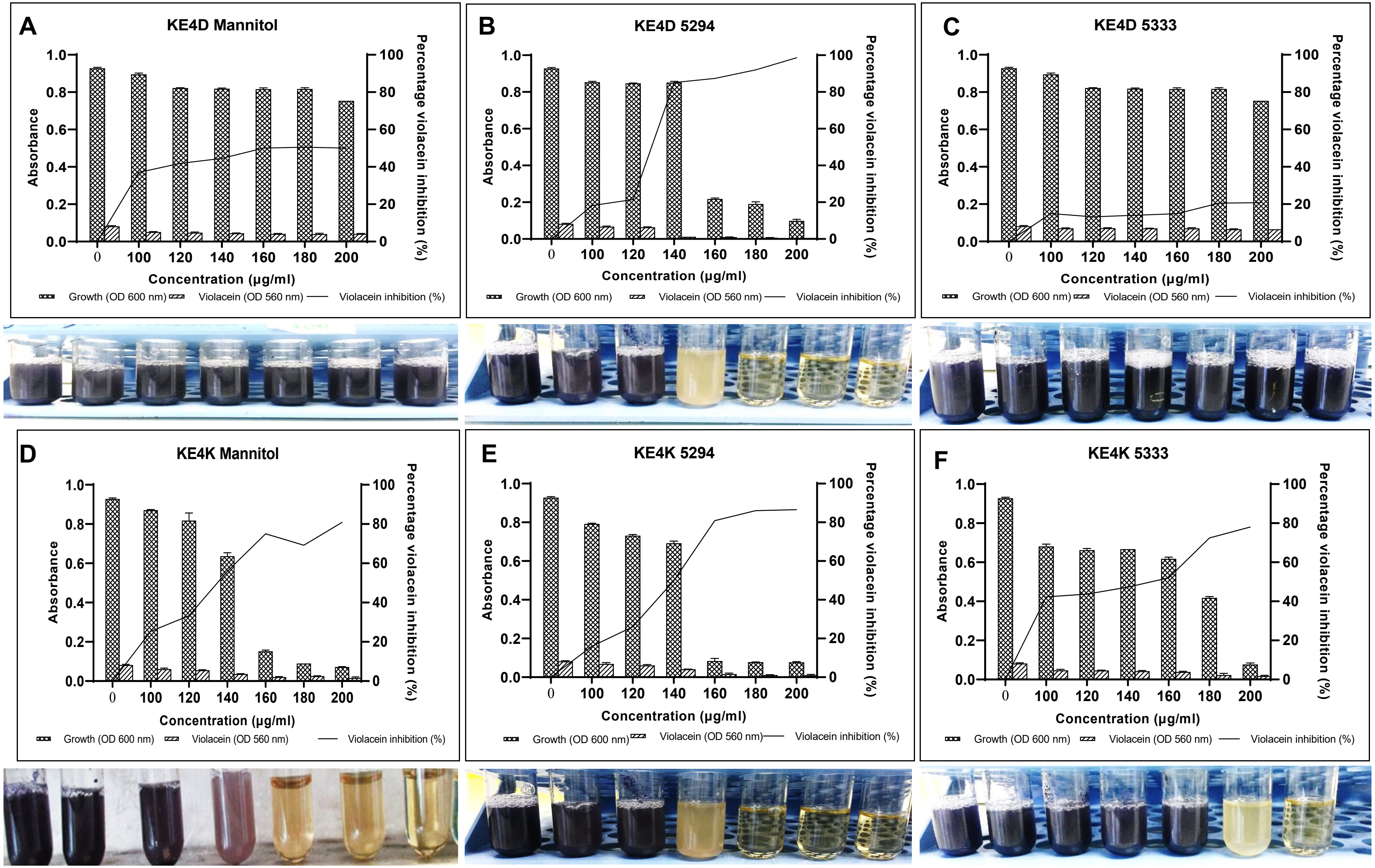

A
KE4D Mannitol
B
KE4D 5294
C
KE4D 5333
D
KE4K Mannitol
E
KE4K 5294
F
KE4K 5333
Absorbance
0.0
0.2
0.4
0.6
0.8
1.0
Percentage violacein inhibition (%)
0
20
40
60
80
100
0
100
120
140
160
180
200
Concentration (µg/ml)
Growth (OD 600 nm)
Violacein (OD 560 nm)
Violacein inhibition (%)

Figure S5. Quantitative analysis of the concentration-dependent, violacein inhibitory effects of *Streptomyces* sp. KE4D and *Bacillus safensis* KE4K silver nanoparticles (AgNPs) biosynthesized from cell-free supernatants obtained following fermentation in three different media: (A) *Streptomyces* sp. KE4D medium Mannitol, (B) *Streptomyces* sp. KE4D medium 5294, (C) *Streptomyces* sp. KE4D medium 5333, (D) *B. safensis* KE4K medium Mannitol, (E) *B. safensis* KE4K medium 5294 and (F) *B. safensis* KE4K medium 5333 at 0-200 µg/ml against *Chromobacterium violaceum* ATCC 12472 Bacterial growth at $OD_{600\ nm}$ and violacein production at $OD_{560\ nm}$ while the solid line graph represents percentage violacein inhibition. Data represents the mean of two independent experiments done in triplicate. Good quorum sensing inhibition was defined as biosynthesised AgNPs exhibiting ≥50% VI with <40% growth inhibition

Table S1. GC-MS analysis of the *Streptomyces* sp. KE4D crude medium Mannitol extract and elucidation of empirical formulas and putative identification of each compound

Retention time	Compound	Area %	Empirical formula	Molecular mass (Da)
12.484	Cyclopropaneacetic acid, 2-hexyl-	2.09	$C_{11}H_{20}O_2$	184
14.265	7R,8R-8-Hydroxy-4-isopropylidene-7-methylbicyclo[	0.81	$C_{15}H_{24}O$	220
15.11	Tetradecanoic acid	0.92	$C_{15}H_{30}O_2$	242
15.813	Tridecanoic acid, 12-methyl-, methyl ester	6.01	$C_{15}H_{30}O_2$	242
16.198	Pentadecanoic acid, methyl ester	0.2	$C_{16}H_{32}O_2$	256
16.337	Pentadecanoic acid	2.14	$C_{16}H_{32}O_2$	256
16.49	Pentadecanoic acid	11.86	$C_{16}H_{32}O_2$	256
17.151	Hexadecanoic acid, methyl ester	1.69	$C_{17}H_{34}O_2$	270
17.796	Pentadecanoic acid, 14-methyl-, methyl ester	1.27	$C_{17}H_{34}O_2$	270
18.728	Hexadecanoic acid	7.83	$C_{16}H_{32}O_2$	256
19.301	Tetradecanoic acid, 5,9,13-trimethyl-, methyl ester	2.13	$C_{18}H_{36}O_2$	284
20.21	Pyrrolo[1,2-a]pyrazine-1,4-dione, hexahydro-3-(2	35.08	$C_{11}H_{18}N_2O_2$	210
20.418	Eicosanoic acid	3.17	$C_{20}H_{40}O_2$	312

Table S2. GC-MS analysis of the *Streptomyces* sp. KE4D crude medium 5294 extract and elucidation of empirical formulas and putative identification of each compound

Retention time	Compound	Area %	Empirical formula	Molecular mass (Da)
3.818	Propanol, 2,2-dimethyl-, acetate	1.32	$C_7H_{14}O_2$	130
7.510	(-)-4-Methylhexanoic acid	0.87	$C_7H_{14}O_2$	130
8.535	Octanoic acid	0.52	$C_9H_{18}O_2$	158
8.977	Dioxane-2,5-dimethanol	2.17	$C_6H_{12}O_4$	148
9.787	4-Methyloctanoic acid	3.01	$C_9H_{18}O_2$	158
11.38	Tetradecanoic acid	1.64	$C_{14}H_{28}O_2$	228
12.553	-Isopropyl-2,4-imidazolidinedione	1.72	$C_6H_{10}N_2O_2$	142
13.552	2,4-Imidazolidinedione, 5-(2-methylpropyl)-, (S)-	5.3	$C_7H_{12}N_2O_2$	156
13.470	Heptadecane, 2,6,10,15-tetramethyl-	3.62	$C_{21}H_{44}$	296
13.996	1-Penten-3-one, 1-(2,6,6-trimethyl-1-cyclohexen-1-yl)	1.84	$C_{14}H_{22}O$	206
13.399	1-Heptadecene	0.61	$C_{17}H_{34}$	238
13.908	Eicosanoic acid	0.87	$C_{20}H_{40}O_2$	312
14.050	3,7-Cyclodecadiene-1-methanol, .alpha.,.alpha.,4,8 tetramethyl	1.22	$C_{15}H_{26}O$	222
14.789	Tetradecanoic acid	0.84	$C_{14}H_{28}O_2$	228
15.511	Eicosane	2.1	$C_{20}H_{42}$	282
15.936	Pentadecanoic acid	0.81	$C_{15}H_{30}O_2$	242
16.073	i-Propyl 12-methyltetradecanoate	6.08	$C_{18}H_{36}O_2$	284
16.160	Pyrrolo[1,2-a]pyrazine-1,4-dione, hexahydro-3-(2-methylpropyl	0.81	$C_{11}H_{18}N_2O_2$	210
17.528	Pyrrolo[1,2-a]pyrazine-1,4-dione, hexahydro-3-(2-methylpropyl	2.00	$C_{11}H_{18}N_2O_2$	210
17.426	l-(+)-Ascorbic acid 2,6-dihexadecanoate	3.11	$C_{38}H_{68}O_8$	652
17.833	Pyrrolo[1,2-a]pyrazine-1,4-dione, hexahydro-3-(2-methylpropyl	2.29	$C_{11}H_{18}N_2O_2$	210
17.959	Dibutyl phthalate	8.33	$C_{16}H_{22}O_4$	278
18.103	l-(+)-Ascorbic acid 2,6-dihexadecanoate	3.96	$C_{38}H_{68}O_8$	652
18.713	Eicosane	2.25	$C_{20}H_{42}$	282
19.658	Eicosanoic acid	2.32	$C_{20}H_{40}O_2$	312

Table S3. GC-MS analysis of the *Streptomyces* sp. KE4D crude medium 5333 extract and elucidation of empirical formulas and putative identification of each compound

Retention time	Compound	Area %	Empirical formula	Molecular mass (Da)
12.603	5-n-Propylhydantoin	5.5	$C_6H_{10}N_2O_2$	142
14.474	Eicosane	0.73	$C_{20}H_{42}$	282
14.680	2,4a-Methanonaphthalen-7(4aH)-one, 1,2,3,4,5,6-hexahydro	0.92	$C_{15}H_{22}O$	218
17.619	Pyrrolo[1,2-a]pyrazine-1,4-dione, hexahydro-3-(2-methylpropyl)-	3.84	$C_{11}H_{18}N_2O_2$	210
17.935	Pyrrolo[1,2-a]pyrazine-1,4-dione, hexahydro-3-(2-methylpropyl)-	2.54	$C_{11}H_{18}N_2O_2$	210
18.077	Dibutyl phthalate	15.54	$C_{16}H_{22}O_4$	278
18.687	Benzonitrile, 3-benzyloxy-	14.76	$C_{14}H_{11}NO$	209

Table S4. GC-MS analysis of the *Bacillus safensis* KE4K crude medium Mannitol extract and elucidation of empirical formulas and putative identification of each compound

Retention time	Compound	Area %	Empirical formula	Molecular mass (Da)
6.347	Isovaline, 3-hydroxy-	18.36	$C_5H_{11}NO_3$	133
6.413	Isovaline, 3-hydroxy-	6.41	$C_5H_{11}NO_4$	134
6.478	2,5-Hexanedione, 3,4-dihydroxy-3,4-dimethyl-	4.03	$C_8H_{14}O_4$	174
7.244	Acetamide, N-(2-methylpropyl)-	4.23	$C_6H_{13}NO$	115
7.973	N(1),N(1)-Diethyl-1,2-butanediamine	1.26	$C_8H_{20}N_2$	144
8.017	Malic Acid	0.47	$C_4H_6O_5$	134
8.084	2,5-Hexanedione, 3,4-dihydroxy-3,4-dimethyl-	0.45	$C_8H_{14}O_4$	174
7.705	Pyrazine, tetramethyl-	6.61	$C_8H_{12}N_2$	136
8.460	Acetamide, N-(2-methylpropyl)-	0.97	$C_6H_{13}NO$	115
8.560	N-(3-Methylbutyl)acetamide	1.32	$C_7H_{15}NO$	129
10.325	l-Alanine, N-pivaloyl-, methyl ester	0.12	$C_9H_{17}NO_3$	187
10.121	5-Methylenehydantoin	0.37	$C_4H_4N_2O_2$	112
10.623	5-Methylenehydantoin	0.9	$C_4H_4N_2O_3$	113
11.902	2-Hydroxy-4-methoxybenzaldehyde, acetate	0.85	$C_{10}H_{10}O_4$	194
13.280	2-(2-Hydroxy-2-methyl-3-oxobutyl)-3,5,6-trimethylpyrazine	2.61	$C_{12}H_{18}N_2O_2$	222
13.165	3-Amino-3-(4-isopropoxy-phenyl)-propionic acid	8.3	$C_{12}H_{17}NO_3$	223
13.460	Pyrido[2,3-d]pyrimidin-5(8H)-one, 2,4,7-trimethyl-	1.38	$C_{10}H_{11}N_3O$	189
13.568	N,3-Diethyl-3-octanamine	0.93	$C_{12}H_{27}N$	185

Table S5. GC-MS analysis of the *Bacillus safensis* KE4K crude medium 5294 extract and elucidation of empirical formulas and putative identification of each compound

Retention time	Compound	Area %	Empirical formula	Molecular mass (Da)
5.375	Butanoic acid, 3-methyl-	23.59	$C_5H_{10}O_2$	102
5.455	Propanamide, N-methyl-	19.62	C_4H_9NO	87
6.482	Isobutyl isothiocyanate	2.17	C_5H_9NS	115
7.670	Pyrazine, tetramethyl-	4.1	$C_8H_{12}N_2$	136
13.470	Heptadecane, 2,6,10,15-tetramethyl-	0.51	$C_{21}H_{44}$	296
13.680	1-Methyl-2-morpholin-4-ylethyl acetate	3.55	$C_9H_{17}NO_3$	187
13.773	N-Methyl-3-hydroxymethylpyrrolidin-2-one	3.5	$C_6H_{11}NO_2$	129
15.513	Eicosane	0.31	$C_{20}H_{42}$	282
17.869	Pyrrolo[1,2-a]pyrazine-1,4-dione, hexahydro-3-(2)methylpropyl	0.41	$C_{11}H_{18}N_2O_2$	210
17.964	Dibutyl phthalate	2.26	$C_{16}H_{22}O_4$	278
18.111	n-Hexadecanoic acid	0.80	$C_{16}H_{32}O_2$	256

Table S6. GC-MS analysis of the *Bacillus safensis* KE4K crude medium 5333 extract and elucidation of empirical formulas and putative identification of each compound

Retention time	Compound	Area %	Empirical formula	Molecular mass (Da)
11.464	Dodecane, 2,6,11-trimethyl-	1.48	$C_{15}H_{32}$	212
13.542	Tricosane-2,4-dione	18.48	$C_{23}H_{44}O_2$	352
15.602	Eicosane	1.97	$C_{20}H_{42}$	282
18.087	Dibutyl phthalate	3.48	$C_{16}H_{22}O_4$	278
29.230	6,19-Cycloandrostane-3,7-diol, 3.beta.-methoxy-	4.00	$C_{20}H_{32}O_3$	320
29.310	Phthalic acid, di(4,4-dimethylpent-2-yl) ester	4.87	$C_{22}H_{34}O_4$	362
29.388	Diisooctyl phthalate	17.17	$C_{24}H_{38}O_4$	390

Table S7. GC-MS analysis of the medium Mannitol fermentation control broth extract and elucidation of empirical formulas and putative identification of each compound

Retention time	Compound	Area %	Empirical formula	Molecular mass (Da)
14.392	Hexanedioic acid, bis(2-methylpropyl) ester	6.84	$C_{14}H_{26}O_4$	258
16.614	1,2-Benzenedicarboxylic acid, bis(2-methylpropyl	57.04	$C_{16}H_{22}O_4$	278
21.650	Dodecanoic acid, isooctyl ester	2.42	$C_{20}H_{40}O_2$	312
23.272	cis-10-Heptadecenoic acid	4.87	$C_{17}H_{32}O_2$	268

Table S8. GC-MS analysis of the medium 5294 fermentation control broth extract and elucidation of empirical formulas and putative identification of each compound

Retention time	Compound	Area %	Empirical formula	Molecular mass (Da)
13.472	Glutaric acid, di(isobutyl) ester	2.40	$C_{13}H_{24}O_4$	244
14.529	Hexanedioic acid, bis(2-methylpropyl) ester	11.29	$C_{14}H_{26}O_4$	258
16.838	1,2-Benzenedicarboxylic acid, bis(2-methylpropyl) ester	66.45	$C_{16}H_{22}O_4$	278
22.059	Dodecanoic acid, isooctyl ester	2.85	$C_{20}H_{40}O_2$	312
23.717	6-Octadecenoic acid, (Z)-	2.51	$C_{18}H_{34}O_2$	282

Table S9. GC-MS analysis of the medium 5333 fermentation control broth extract and elucidation of empirical formulas and putative identification of each compound

Retention time	Compound	Area %	Empirical formula	Molecular mass (Da)
11.419	Propanoic acid, 2-methyl-, 3-hydroxy-2,4,4-trimethylpentyl	1.50	$C_{12}H_{24}O_3$	216
11.658	2,4-Imidazolidinedione, 5-methyl-	4.88	$C_4H_6N_2O_2$	114
12.853	5-Isopropyl-2,4-imidazolidinedione	3.10	$C_6H_{10}N_2O_2$	142
13.510	Glutaric acid, isobutyl undecyl ester	1.34	$C_{20}H_{38}O_4$	342
13.881	2,4-Imidazolidinedione, 5-(2-methylpropyl)-, (S)-	15.59	$C_7H_{12}N_2O_2$	156
14.567	Hexanedioic acid, bis(2-methylpropyl) ester	6.16	$C_{14}H_{26}O_4$	258
16.878	1,2-Benzenedicarboxylic acid, bis(2-methylpropyl)	42.95	$C_{16}H_{22}O_4$	278
